# A Genome-Wide Association Meta-Analysis of Circulating Sex Hormone–Binding Globulin Reveals Multiple Loci Implicated in Sex Steroid Hormone Regulation

**DOI:** 10.1371/journal.pgen.1002805

**Published:** 2012-07-19

**Authors:** Andrea D. Coviello, Robin Haring, Melissa Wellons, Dhananjay Vaidya, Terho Lehtimäki, Sarah Keildson, Kathryn L. Lunetta, Chunyan He, Myriam Fornage, Vasiliki Lagou, Massimo Mangino, N. Charlotte Onland-Moret, Brian Chen, Joel Eriksson, Melissa Garcia, Yong Mei Liu, Annemarie Koster, Kurt Lohman, Leo-Pekka Lyytikäinen, Ann-Kristin Petersen, Jennifer Prescott, Lisette Stolk, Liesbeth Vandenput, Andrew R. Wood, Wei Vivian Zhuang, Aimo Ruokonen, Anna-Liisa Hartikainen, Anneli Pouta, Stefania Bandinelli, Reiner Biffar, Georg Brabant, David G. Cox, Yuhui Chen, Steven Cummings, Luigi Ferrucci, Marc J. Gunter, Susan E. Hankinson, Hannu Martikainen, Albert Hofman, Georg Homuth, Thomas Illig, John-Olov Jansson, Andrew D. Johnson, David Karasik, Magnus Karlsson, Johannes Kettunen, Douglas P. Kiel, Peter Kraft, Jingmin Liu, Östen Ljunggren, Mattias Lorentzon, Marcello Maggio, Marcello R. P. Markus, Dan Mellström, Iva Miljkovic, Daniel Mirel, Sarah Nelson, Laure Morin Papunen, Petra H. M. Peeters, Inga Prokopenko, Leslie Raffel, Martin Reincke, Alex P. Reiner, Kathryn Rexrode, Fernando Rivadeneira, Stephen M. Schwartz, David Siscovick, Nicole Soranzo, Doris Stöckl, Shelley Tworoger, André G. Uitterlinden, Carla H. van Gils, Ramachandran S. Vasan, H.-Erich Wichmann, Guangju Zhai, Shalender Bhasin, Martin Bidlingmaier, Stephen J. Chanock, Immaculata De Vivo, Tamara B. Harris, David J. Hunter, Mika Kähönen, Simin Liu, Pamela Ouyang, Tim D. Spector, Yvonne T. van der Schouw, Jorma Viikari, Henri Wallaschofski, Mark I. McCarthy, Timothy M. Frayling, Anna Murray, Steve Franks, Marjo-Riitta Järvelin, Frank H. de Jong, Olli Raitakari, Alexander Teumer, Claes Ohlsson, Joanne M. Murabito, John R. B. Perry

**Affiliations:** 1 Section of Preventive Medicine and Epidemiology, Boston University School of Medicine, Boston, Massachusetts, United States of America; 2 Section of Endocrinology, Diabetes, and Nutrition, Boston University School of Medicine, Boston, Massachusetts, United States of America; 3 National Heart, Lung, and Blood Institute's The Framingham Heart Study, Framingham, Massachusetts, United States of America; 4 Institute for Clinical Chemistry and Laboratory Medicine, University Medicine, Ernst-Moritz-Arndt University of Greifswald, Greifswald, Germany; 5 Department of Medicine and Department of Obstetrics and Gynecology, The University of Alabama at Birmingham, Birmingham, Alabama, United States of America; 6 Department of Medicine, Johns Hopkins University, Baltimore, Maryland, United States of America; 7 Department of Clinical Chemistry, Fimlab Laboratories, Tampere University Hospital and University of Tampere School of Medicine, Tampere, Finland; 8 Wellcome Trust Centre for Human Genetics, University of Oxford, Oxford, United Kingdom; 9 Department of Biostatistics, Boston University School of Public Health, Boston, Massachusetts, United States of America; 10 Department of Public Health, Indiana University School of Medicine, Indianapolis, Indiana, United States of America; 11 Melvin and Bren Simon Cancer Center, Indiana University, Indianapolis, Indiana, United States of America; 12 University of Texas Health Sciences Center at Houston, Houston, Texas, United States of America; 13 Oxford Centre for Diabetes, Endocrinology, and Metabolism, University of Oxford, Oxford, United Kingdom; 14 Department of Twin Research and Genetic Epidemiology, King's College London, London, United Kingdom; 15 Julius Center for Health Sciences and Primary Care, University Medical Center Utrecht, Utrecht, The Netherlands; 16 Program on Genomics and Nutrition and the Center for Metabolic Disease Prevention, School of Public Health, University of California Los Angeles, Los Angeles, California, United States of America; 17 Center for Bone and Arthritis Research, Institute of Medicine, Sahlgrenska Academy, University of Gothenburg, Gothenburg, Sweden; 18 Laboratory of Epidemiology, Demography, and Biometry, National Institute on Aging, Bethesda, Maryland, United States of America; 19 Wake Forest University School of Medicine, Winston-Salem, North Carolina, United States of America; 20 Department of Epidemiology and Prevention, Division of Public Health Sciences, Wake Forest University Health Sciences, Winston-Salem, North Carolina, United States of America; 21 Laboratory of Epidemiology, Demography, and Biometry, National Institute on Aging, Bethesda, Maryland, United States of America; 22 Institute of Genetic Epidemiology, Helmholtz Zentrum München, Neuherberg, Germany; 23 Program in Molecular and Genetic Epidemiology, Department of Epidemiology, Harvard School of Public Health, Boston, Massachusetts, United States of America; 24 Channing Laboratory, Department of Medicine, Brigham and Women's Hospital, and Harvard Medical School, Boston, Massachusetts, United States of America; 25 Department of Internal Medicine, Erasmus MC, Rotterdam, The Netherlands; 26 Netherlands Consortium of Healthy Aging, Rotterdam, The Netherlands; 27 Genetics of Complex Traits, Peninsula Medical School, University of Exeter, Exeter, United Kingdom; 28 Institute of Diagnostics, University of Oulu, Oulu, Finland; 29 Department of Obstetrics and Gynecology, University Hospital of Oulu, Oulu, Finland; 30 National Institute for Health and Welfare and Institute of Health Sciences, University of Oulu, Oulu, Finland; 31 Geriatric Unit, Azienda Sanitaria di Firenze, Florence, Italy; 32 Department of Prosthetic Dentistry, Gerostomatology, and Dental Materials, University of Greifswald, Greifswald, Germany; 33 Experimental and Clinical Endocrinology, University of Lübeck, Lübeck, Germany; 34 Cancer Research Center of Lyon, INSERM U1052, Lyon, France; 35 Department of Epidemiology and Biostatistics, School of Public Health, Imperial College, London, United Kingdom; 36 California Pacific Medical Center, San Francisco, California, United States of America; 37 Longitudinal Studies Section, Clinical Research Branch, National Institute on Aging, Baltimore, Maryland, United States of America; 38 Division of Biostatistics and Epidemiology, University of Massachusetts, Amherst, Massachusetts, United States of America; 39 Department of Epidemiology, Harvard School of Public Health, Boston, Massachusetts, United States of America; 40 Department of Epidemiology, Erasmus MC, Rotterdam, The Netherlands; 41 Interfaculty Institute for Genetics and Functional Genomics, University of Greifswald, Greifswald, Germany; 42 Research Unit of Molecular Epidemiology, Helmholtz Zentrum München, Neuherberg, Germany; 43 Hannover Unified Biobank, Hannover Medical School, Hannover, Germany; 44 Hebrew SeniorLife Institute for Aging Research and Harvard Medical School, Boston, Massachusetts, United States of America; 45 Clinical and Molecular Osteoporosis Research Unit, Department of Clinical Sciences and Department of Orthopaedics, Lund University, Malmö, Sweden; 46 Institute for Molecular Medicine Finland (FIMM), University of Helsinki, Helsinki, Finland; 47 Department of Chronic Disease Prevention, National Institute for Health and Welfare, Helsinki, Finland; 48 Program in Molecular and Genetic Epidemiology, Department of Epidemiology, Harvard School of Public Health, Boston, Massachusetts, United States of America; 49 Women's Health Initiative Clinical Coordinating Center, Division of Public Health Sciences, Fred Hutchinson Cancer Research Center, Seattle, Washington, United States of America; 50 Department of Medical Sciences, University of Uppsala, Uppsala, Sweden; 51 Department of Internal Medicine and Biomedical Sciences, Section of Geriatrics, University of Parma, Parma, Italy; 52 Institute for Community Medicine, University of Greifswald, Greifswald, Germany; 53 University of Pittsburgh, Pittsburgh, Pennsylvania, United States of America; 54 Gene Environment Initiative, Program in Medical and Population Genetics, Broad Institute of Harvard and MIT, Boston, Massachusetts, United States of America; 55 Department of Biostatistics, University of Washington, Seattle, Washington, United States of America; 56 Medical Genetics Institute, Cedars-Sinai Medical Center, Los Angeles, California, United States of America; 57 Medizinische Klinik and Poliklinik IV, Ludwig-Maximilians University, Munich, Germany; 58 Division of Public Health Sciences, Fred Hutchinson Cancer Research Center, Seattle, Washington, United States of America; 59 Brigham and Women's Hospital, Harvard Medical School, Boston, Massachusetts, United States of America; 60 Cardiovascular Health Research Unit, Department of Epidemiology, University of Washington, Seattle, Washington, United States of America; 61 Human Genetics, Wellcome Trust Sanger Institute, Hinxton, United Kingdom; 62 Institute of Epidemiology II, Helmholtz Zentrum München, Neuherberg, Germany; 63 Department of Obstetrics and Gynaecology, Ludwig-Maximilians-University, Munich, Germany; 64 Institute of Epidemiology I, Helmholtz Zentrum München, Neuherberg, Germany; 65 Institute of Medical Informatics, Biometry, and Epidemiology, Ludwig-Maximilians-Universität, Munich, Germany; 66 Klinikum Großhadern, Munich, Germany; 67 Discipline of Genetics, Faculty of Medicine, Memorial University of Newfoundland, St. John's, Newfoundland, Canada; 68 Division of Cancer Epidemiology and Genetics, National Cancer Institute, National Institutes of Health, Bethesda, Maryland, United States of America; 69 Department of Clinical Physiology, Tampere University Hospital and University of Tampere School of Medicine, Tampere, Finland; 70 Program on Genomics and Nutrition, Department of Epidemiology, University of California Los Angeles, Los Angeles, California, United States of America; 71 Division of Cardiology, Johns Hopkins Bayview Medical Center, Baltimore, Maryland, United States of America; 72 Department of Medicine, Turku University Hospital and University of Turku, Turku, Finland; 73 Oxford Centre for Diabetes, Endocrinology, and Metabolism, University of Oxford, Churchill Hospital, Oxford, United Kingdom; 74 Oxford NIHR Biomedical Research Centre, Churchill Hospital, Oxford, United Kingdom; 75 Institute of Reproductive and Developmental Biology, Imperial College London, London, United Kingdom; 76 Department of Biostatistics and Epidemiology, School of Public Health, MRC-HPA Centre for Environment and Health, Faculty of Medicine, Imperial College London, London, United Kingdom; 77 Institute of Health Sciences, University of Oulu, Oulu, Finland; 78 Biocenter Oulu, University of Oulu, Oulu, Finland; 79 National Institute of Health and Welfare, University of Oulu, Oulu, Finland; 80 Department of Clinical Physiology and Nuclear Medicine, Turku University Hospital and Research Centre of Applied and Preventive Cardiovascular Medicine, University of Turku, Turku, Finland; 81 Section of General Internal Medicine, Boston University School of Medicine, Boston, Massachusetts, United States of America; Georgia Institute of Technology, United States of America

## Abstract

Sex hormone-binding globulin (SHBG) is a glycoprotein responsible for the transport and biologic availability of sex steroid hormones, primarily testosterone and estradiol. SHBG has been associated with chronic diseases including type 2 diabetes (T2D) and with hormone-sensitive cancers such as breast and prostate cancer. We performed a genome-wide association study (GWAS) meta-analysis of 21,791 individuals from 10 epidemiologic studies and validated these findings in 7,046 individuals in an additional six studies. We identified twelve genomic regions (SNPs) associated with circulating SHBG concentrations. Loci near the identified SNPs included *SHBG* (rs12150660, 17p13.1, p = 1.8×10^−106^), *PRMT6* (*rs17496332, 1p13.3*, p = *1.4*×*10^−11^*), *GCKR* (*rs780093*, *2p23.3*, p = *2.2*×*10^−16^*), *ZBTB10* (*rs440837*, *8q21.13*, p = *3.4*×*10^−09^*), *JMJD1C* (*rs7910927*, *10q21.3*, p = *6.1*×*10^−35^*), *SLCO1B1* (*rs4149056*, *12p12.1*, p = *1.9*×*10^−08^*), *NR2F2* (*rs8023580*, *15q26.2*, p = *8.3*×*10^−12^*), *ZNF652* (*rs2411984*, *17q21.32*, p = *3.5*×*10^−14^*), *TDGF3* (*rs1573036*, *Xq22.3*, p = *4.1*×*10^−14^*), *LHCGR* (*rs10454142*, *2p16.3*, p = *1.3*×*10^−07^*), *BAIAP2L1* (*rs3779195*, *7q21.3*, p = *2.7*×*10^−08^*), and *UGT2B15* (*rs293428*, *4q13.2*, p = *5.5*×*10^−06^*). These genes encompass multiple biologic pathways, including hepatic function, lipid metabolism, carbohydrate metabolism and T2D, androgen and estrogen receptor function, epigenetic effects, and the biology of sex steroid hormone-responsive cancers including breast and prostate cancer. We found evidence of sex-differentiated genetic influences on SHBG. In a sex-specific GWAS, the loci 4q13.2-*UGT2B15* was significant in men only (men p = 2.5×10^−08^, women p = 0.66, heterogeneity p = 0.003). Additionally, three loci showed strong sex-differentiated effects: 17p13.1-*SHBG* and Xq22.3-*TDGF3* were stronger in men, whereas 8q21.12-*ZBTB10* was stronger in women. Conditional analyses identified additional signals at the *SHBG* gene that together almost double the proportion of variance explained at the locus. Using an independent study of 1,129 individuals, all SNPs identified in the overall or sex-differentiated or conditional analyses explained ∼15.6% and ∼8.4% of the genetic variation of SHBG concentrations in men and women, respectively. The evidence for sex-differentiated effects and allelic heterogeneity highlight the importance of considering these features when estimating complex trait variance.

## Author Summary

Sex hormone-binding globulin (SHBG) is the key protein responsible for binding and transporting the sex steroid hormones, testosterone and estradiol, in the circulatory system. SHBG regulates their bioavailability and therefore their effects in the body. SHBG has been linked to chronic diseases including type 2 diabetes and to hormone-sensitive cancers such as breast and prostate cancer. SHBG concentrations are approximately 50% heritable in family studies, suggesting SHBG concentrations are under significant genetic control; yet, little is known about the specific genes that influence SHBG. We conducted a large study of the association of SHBG concentrations with markers in the human genome in ∼22,000 white men and women to determine which loci influence SHBG concentrations. Genes near the identified genomic markers in addition to the *SHBG* protein coding gene included *PRMT6*, *GCKR*, *ZBTB10*, *JMJD1C*, *SLCO1B1*, *NR2F2*, *ZNF652*, *TDGF3*, *LHCGR*, *BAIAP2L1*, and *UGT2B15*. These genes represent a wide range of biologic pathways that may relate to SHBG function and sex steroid hormone biology, including liver function, lipid metabolism, carbohydrate metabolism and type 2 diabetes, and the development and progression of sex steroid hormone-responsive cancers.

## Introduction

Sex hormone-binding globulin (SHBG) is a protein secreted mainly by the liver that binds to the sex steroids, testosterone, dihydrotestosterone, and estradiol, transports them in the circulation, and influences their action in target tissues by regulating their bioavailability. SHBG thereby influences the expression of sex hormone sensitive phenotypes including sexual characteristics and reproductive function in men and women [1]. In addition to regulating sex steroid hormone effects, SHBG may exert independent effects through its own receptor [2]. Variation in SHBG concentration has also been associated with various chronic diseases including cancers [3], polycystic ovary syndrome (PCOS) [4], [5] and type 2 diabetes (T2D) [6], [7]. Although SHBG is estimated to have a heritable component (∼50%) [8], little is known about the genetic regulation of SHBG. Polymorphisms at the *SHBG* gene locus have been associated with SHBG concentrations [9], [10], but much remains unknown about specific genetic variants that may determine circulating SHBG concentrations. Identifying genetic factors that influence SHBG may provide insights into the biology of sex steroid hormone regulation, metabolism and tissue effects that underlie their relationship with chronic diseases such as T2D as well as hormone-sensitive cancers such as breast and prostate cancer.

## Results

**Figure 1:**
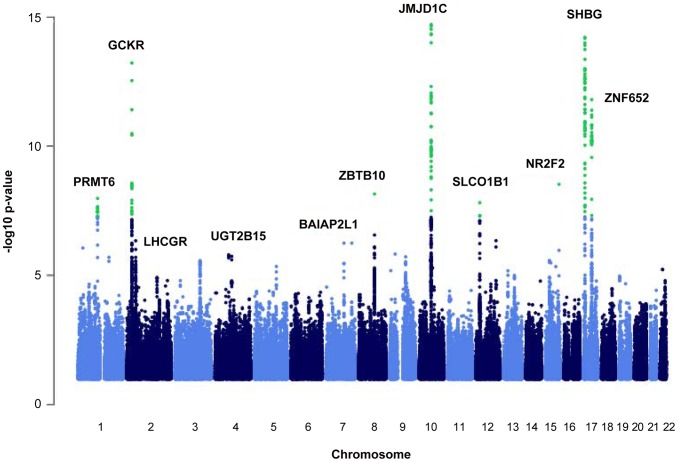
Manhattan plot of the autosomal SNPs identified in the GWA meta-analysis. The Manhattan plot depicts the SNPs identified in the GWAS analysis labeled with the nearest gene on the plot. The SNP identified on the X chromosome, rs1573036, at Xq22.3, is not included in this figure.

We identified nine loci associated with SHBG concentrations at the genome-wide significance threshold of p = 5×10^−8^ (Table 1 and Figure 1) in a genome-wide association study (GWAS) meta-analysis of circulating SHBG concentrations in 21,791 men and women from 10 studies (Table S1). All nine lead SNPs at these loci had effects in the same direction (seven with p<0.05) in the validation dataset of 7,046 men and women from six additional studies (Table S2). The strongest association was within the *SHBG* locus (rs12150660, p = 2×10^−106^). Together, these nine lead SNPs explained 7.2% of the genetic variance (assuming 50% heritability) in SHBG concentrations.

**Table 1:** SNPs representing loci associated with circulating SHBG concentrations.

									Discovery Samples			Discovery+Follow-up		
Index SNP	Analysis	Region	Nr Gene	Chr	Position	Effect Allele	Other Allele	EAF	Beta	SE	p	Beta	SE	p
rs17496332	Main	1p13.3	PRMT6	1	107347898	a	g	0.67	−0.026	0.0046	1.0E-08	−0.028	0.0041	1.4E-11
rs780093	Main	2p23.3	GCKR	2	27596107	t	c	0.40	−0.033	0.0043	5.8E-14	−0.032	0.0039	2.2E-16
rs440837	Main	8q21.13	ZBTB10	8	81624529	a	g	0.78	−0.030	0.0052	6.7E-09	−0.028	0.0047	3.4E-09
rs7910927	Main	10q21.3	JMJD1C	10	64808916	t	g	0.51	−0.044	0.0043	7.4E-25	−0.048	0.0039	6.1E-35
rs4149056	Main	12p12.1	SLCO1B1	12	21222816	t	c	0.82	0.032	0.0057	1.5E-08	0.029	0.0052	1.9E-08
rs8023580	Main	15q26.2	NR2F2	15	94509295	t	c	0.72	−0.029	0.0049	2.8E-09	−0.03	0.0044	8.3E-12
rs12150660	Main	17p13.1	SHBG	17	7462640	t	g	0.24	0.100	0.0053	1.2E-79	0.103	0.0047	1.8E-106
rs2411984	Main	17q21.32	ZNF652	17	44800750	a	g	0.28	0.034	0.0049	1.5E-12	0.033	0.0044	3.5E-14
rs1573036	Main	Xq22.3	TDGF3	23	109706724	t	c	0.39	0.031	0.0043	5.1E-13	0.028	0.0037	4.1E-14
rs10454142	Conditional	2p16.3	LHCGR	2	48499903	t	c	0.69	0.026	0.0047	2.8E-08	0.023	0.0044	1.3E-07
rs3779195	Conditional	7q21.3	BAIAP2L1	7	97831298	a	t	0.17	−0.033	0.0057	1.2E-08	−0.028	0.0051	2.7E-08
rs293428	Sex-specific	4q13.2	UGT2B15	4	69626371	a	g	0.69	−0.023	0.0047	1.6E-06	−0.019	0.0042	5.5E-06

	Discovery Men			Discovery+Follow-up Men			Discovery Women			Discovery+Follow-up Women			Heterogeneity	
Index SNP	Beta	SE	p	Beta	SE	p	Beta	SE	p	Beta	SE	p	p	Sex
rs17496332	−0.027	0.0054	9.6E-07	−0.027	0.0051	1.5E-07	−0.02	0.008	0.003	−0.029	0.0067	1.8E-05	0.79	Females
rs780093	−0.029	0.0052	1.8E-08	−0.026	0.0049	7.0E-08	−0.04	0.0076	1.3E-07	−0.041	0.0063	8.6E-11	0.07	Females
rs440837	−0.021	0.0062	0.0009	−0.019	0.0058	0.001	−0.049	0.0093	1.2E-07	−0.042	0.0078	7.2E-08	0.02	Females
rs7910927	−0.049	0.0051	5.3E-22	−0.050	0.0048	1.2E-25	−0.038	0.0075	6.4E-07	−0.046	0.0063	1.7E-13	0.63	Males
rs4149056	0.028	0.0067	0.00003	0.027	0.0063	1.5E-05	0.049	0.0103	0.000002	0.037	0.0086	1.7E-05	0.36	Females
rs8023580	−0.024	0.0057	0.00002	−0.025	0.0054	5.1E-06	−0.038	0.0087	0.00001	−0.038	0.0071	7.8E-08	0.13	Females
rs12150660	0.106	0.0063	1.8E-63	0.110	0.0058	3.7E-80	0.085	0.0094	1.8E-19	0.087	0.0077	5.8E-30	0.02	Males
rs2411984	0.034	0.0058	5.9E-09	0.034	0.0054	2.3E-10	0.032	0.0084	0.0001	0.029	0.007	3.2E-05	0.54	Males
rs1573036	0.04	0.0048	9.1E-17	0.035	0.0043	2.8E-16	0.012	0.0083	0.15	0.016	0.007	0.02	0.02	Males
rs10454142														
rs3779195														
rs293428	−0.032	0.0056	1.5E-08	−0.029	0.0053	2.5E-08	−0.005	0.0085	0.57	−0.003	0.0071	0.66	0.003	Males

All SNPs are on the+strand and positions are based on build 36. EAF = ‘effect allele frequency’. Beta units are per-allele effect estimates in natural log transformed nmol/L. Sex column gives the sex with the largest per-allele beta estimate. Missing values for conditional SNPs as sex-specific conditional analysis was not performed.

**Figure 2:**
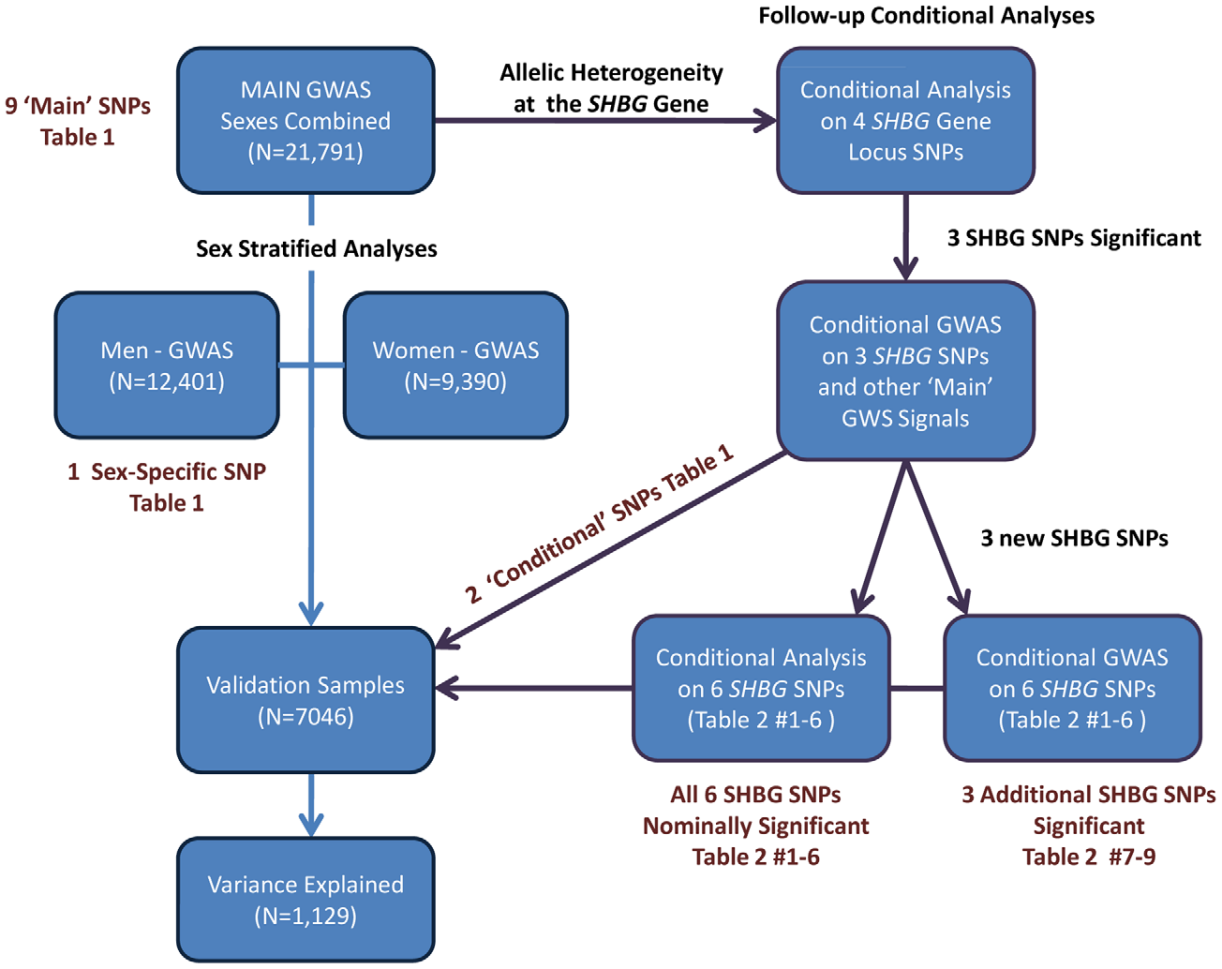
Summary of the analytic plan.

We next performed a series of additional analyses to explain more of the phenotypic variance (Figure 2). First, we hypothesized that genetic effects may be different in men and women, as SHBG concentrations are >50% higher in females than males, and may be differentially regulated between sexes. In a sex stratified analysis, three of the nine loci showed evidence of sex-differentiated effects at p<0.02 when we would not expect any signals to have reached this level of significance by chance. The associations at the 17p13.1-*SHBG* and Xq22.3 loci were stronger in males whereas the association at the 8q21.13 locus was stronger in females. To investigate the apparent differential sex effect for the X chromosome further we ran a recessive regression model for the X chromosome SNP rs1573036 in women in the Framingham Heart Study and found no association with SHBG suggesting the sex-differentiated effect is not the result of a recessive inheritance pattern. Sex stratified GWAS identified one novel signal in men, which showed no association in women (4q13.2: men p = 2.5×10^−8^, women p = 0.66, heterogeneity p = 0.003).

**Figure 3:**
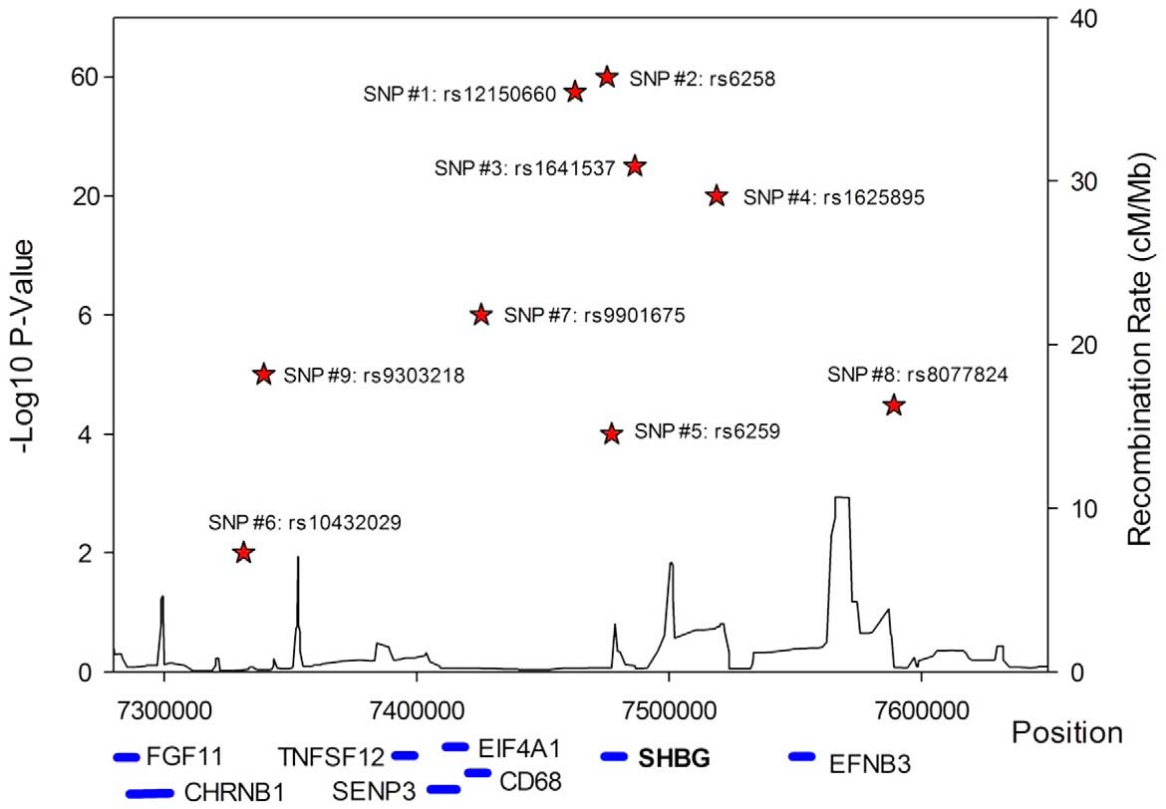
Allelic heterogeneity at the *SHBG* gene locus. There was significant allelic heterogeneity at the SHBG gene locus. The nine independent signals identified in the SHBG gene are shown in relation to their position within the gene. All positions based on build 36. Not all genes are shown.

A series of conditional analyses were performed to identify statistically independent signals. At the *SHBG* locus, three apparently independent additional signals separate from the main index SNP were observed, based on low (r^2^<0.05) pairwise correlations in HapMap (rs6258 p = 2.7×10^−46^, rs1625895 p = 1.2×10^−14^ and rs3853894 p = 2.5×10^−11^). A series of iterative conditional analyses (Table 2) involving SNPs at the *SHBG* locus generated a final regression model including six statistically independent *SHBG* SNPs. Four of these SNPs (#1–4 Table 2) retained GWS when conditioned against the other five, and two were nominally associated (SNP#5 p = 0.0001, SNP#6 p = 0.01). Re-running the GWAS meta-analysis adjusting for these six SNPs revealed evidence for three additional statistically independent (pairwise HapMap r^2^<0.01) signals at the *SHBG* locus (SNP#7 p = 1.5×10^−7^, SNP#8 p = 4.6×10^−5^, SNP#9 p = 9.9×10^−6^) (Figure 3). There were also two additional *trans* signals located at 2p16.3 and 7q21.3 (Table 1). Although the 2p16.3 signal dropped below GWS when combined with follow-up samples (p = 1×10^−7^), the index SNP at 2p16.3 is ∼300 kb away from a strong candidate gene, the luteinizing hormone receptor gene (*LHCGR*).

**Table 2:** Statistically independent signals at the *SHBG* gene locus.

SNP #	Model	Conditioned On SNP #	SNP	Position	Effect Allele	Other Allele	EAF	Beta	SE	p-value	Discovery p-value	Discovery Beta
1	Full model	2–6	rs12150660	7462640	t	g	0.24	0.082	0.005	1.89E-55	1.19E-79	0.10
2	Full model	1,3–6	rs6258	7475403	t	c	0.02	−0.272	0.017	1.03E-60	2.69E-46	−0.2613
3	Full model	1–2,4–6	rs1641537	7486446	t	c	0.14	−0.064	0.006	1.20E-24	8.19E-39	−0.0814
4	Full model	1–3,5–6	rs1625895	7518840	t	c	0.12	−0.06	0.006	1.75E-21	1.17E-14	−0.052
5	Full model	1–4,6	rs6259	7477252	a	g	0.11	0.026	0.007	0.0001	1.46E-07	0.0372
6	Full model	1–5	rs10432029	7331393	a	g	0.79	0.0136	0.006	0.01	7.52E-16	0.0446
7	Conditional	1–6	rs9901675	7425536	a	g	0.05	−0.057	0.01	1.46E-07	5.2E-12	−0.07
8	Conditional	1–6	rs8077824	7588951	a	g	0.02	0.075	0.018	4.58E-05	0.01	0.0451
9	Conditional	1–6	rs9303218	7339386	t	c	0.77	0.026	0.006	9,89E-06	1.21E-11	0.0344

All SNPs are on the+strand and positions are based on build 36. EAF = ‘effect allele frequency’. Beta units are per-allele effect estimates in natural log transformed nmol/L. ‘Full model’ SNPs were all included in a single regression model, where the effect estimates for each SNP are adjusted for the effect of the others in the model.

‘Conditional’ SNPs are SNPs with low pair-wise LD (HapMap r^2^<0.01) that were identified after conditioning on the full model SNPs.

The majority of pair-wise correlations for the nine *SHBG* locus SNPs highlighted by our conditional analyses showed very low HapMap r^2^ values. However, the pairwise D′ values are often high (Table S3) indicating that no or few recombination events have occurred between some SNPs, and that combinations of SNPs may be tagging un-typed variants on a common haplotype. To investigate this possibility, we performed more extensive analyses in a single study (NFBC1966, n = 4467). We used a denser set of SNPs imputed from the June 2011 version of the 1000 Genomes data and performed model selection analyses. Model selection identifies a set of SNPs that best explain phenotypic variation, while simultaneously penalizing each SNP included in this set, and therefore correlated SNPs tend to be excluded from the final model. These analyses consistently included at least seven SNPs in the model, although it is hard to estimate the false-negative rate of using the reduced sample size. While we are underpowered to accurately pinpoint the exact number of independent signals, these analyses support the results of the conditional analysis and suggest that multiple variants at the *SHBG* locus are independently associated with SHBG concentrations.

Data from an independent study, the InCHIANTI study, was used to calculate the proportion of genetic variance in SHBG concentrations explained when accounting for sex specific effects, the multiple signals of association at the *SHBG* locus, and the additional *trans* signals identified post conditional analysis. In men and women we explained ∼15.6% and ∼8.4% of the heritable component respectively. The *SHBG* locus accounted for ∼10% and ∼6.6% of the genetic variation in men and women respectively with the lead SNP in isolation accounting for ∼7.8% and ∼3.3% of the variation in men and women, respectively.

We identified genes near the associated SNPs and explored their biologic relevance to SHBG. The genes associated with identified SNPs included the *SHBG* locus (rs12150660, 17p13.1, p = 1.8×10^−106^), *PRMT6* (rs17496332, 1p13.3, p = 1.4×10^−11^), *GCKR* (rs780093, 2p23.3, p = 2.2×10^−16^), *ZBTB10* (rs440837, 8q21.13, p = 3.4×10^−09^), *JMJD1C* (rs7910927, 10q21.3, p = 6.1×10^−35^), *SLCO1B1* (rs4149056, 12p12.1, p = 1.9×10^−08^), *NR2F2* (rs8023580, 15q26.2, p = 8.3×10^−12^), *ZNF652* (rs2411984, 17q21.32, p = 3.5×10^−14^), *TDGF3* (rs1573036, Xq22.3, p = 4.1×10^−14^), *LHCGR* (rs10454142, 2p16.3, p = 1.3×10^−07^), *BAIAP2L1* (rs3779195, 7q21.3, p = 2.7×10^−08^), and *UGT2B15* (rs293428, 4q13.2, p = 5.5×10^−06^) (Figure 1).

We used the online tool STRING (www.string-db.org) to perform pathway analyses to explore possible interactions between the *SHBG* gene and the proteins encoded by the 11 most plausible genes nearest the 11 SNPs listed above. There was an interaction noted between *GCKR* and *JMJD1C* which were associated with the lipoprotein fractions VLDL and HDL, respectively [11]. In an expanded analysis, we assessed protein interactions among *SHBG* and 67 genes within 500 kb of our 11 identified SNPs and uncovered additional protein interaction pathways. An interaction between two proteins encoded by *GTF2A1L* and *STON1* was found; these proteins are co-expressed in testicular germ cells in the mouse [12]. An interaction between *LHCGR* and *BRI3* encoded proteins that are associated with the G-protein coupled receptor complex in the human luteinizing hormone receptor was also identified [13]. Finally, an interaction between *LHCGR* and *IAPP* (amylin) proteins which are components of a ligand/G-protein receptor/G-protein alpha subunit complex was found (database: www.reactome.com).

Targeted analysis of two strong candidate genes, hepatocyte nuclear factor-4α (*HNF4*α) and peroxisome-proliferating receptor γ (*PPARγ*) did not identify any SNPs at *HNF4*α but did identify one SNP, rs2920502, at *PPARγ* that reached statistical significance (p = 9.9×10^−5^) and a second SNP at *PPARγ*, rs13081389, that reached nominal significance (p = 0.01).

## Discussion

In total, we identified 12 genomic regions associated with circulating SHBG concentrations, including extensive allelic heterogeneity at the *SHBG* locus itself. Conditional meta-analyses carried out at the *SHBG* locus, identified nine genome-wide significant SNPs with low correlation (r^2^<0.01) between them. Two of these signals (rs6258 [10] and rs6259) are missense variants and two are low frequency variants (MAF ∼2%). Furthermore, rs12150660 is highly correlated (r^2^>0.95) [10] with a pentanucleotide repeat, which affects *SHBG* expression *in-vitro* [14]. To our knowledge, the magnitude of secondary signals observed at this locus are the largest seen for any complex trait.

The proportion of genetic variance in SHBG serum concentrations explained when accounting for sex specific effects, the multiple signals of association at the *SHBG* locus, and the additional *trans* signals identified post conditional analysis was ∼15.6% in men and ∼8.4% in women. The *SHBG* locus accounted for ∼10% and ∼6.6% of the genetic variance in men and women, respectively, with the lead SNP explaining most of the genetic variation at ∼7.8% for men and ∼3.3% for women. Thus additional signals at the *SHBG* locus identified through conditional analyses approximately doubled the variance of the trait explained. While we provide evidence for multiple variants associated with SHBG concentrations, further studies are needed to pinpoint the causal loci and functional variants. For the 11 regions outside the *SHBG* locus, most have biologically plausible related genes within 300 kb.

### Biology of Plausible Genes near Identified SNPs

Several genes near the identified SNPs regulate sex steroid production and function. The *NR2F2* locus (15q26.2) encodes a nuclear receptor important in testicular Leydig cell function, the primary source of gonadal testosterone production [15], and has been linked to male infertility [16]. *NR2F2* has also been associated with estrogen receptor alpha (ERα) signaling and may influence hormone responsivity in breast cancer [17]. *PRMT6* (1p13.3) also encodes a nuclear receptor regulatory protein that mediates estrogen signaling as a co-activator of the estrogen receptor [18]. *LHCGR* (2p16.3) encodes the luteinizing hormone receptor which was associated with polycystic ovary syndrome (PCOS) in a recent GWAS [19], [20]. PCOS is both a reproductive and metabolic disorder characterized by higher testosterone serum concentrations as well as an increased prevalence of obesity, insulin resistance, and T2D in women. Inappropriate secretion of luteinizing hormone leads to increased ovarian production of testosterone. Coincident lower SHBG concentrations contribute to increased bioavailable testosterone concentrations and the expression of both reproductive and metabolic phenotypes in PCOS [21], [22], [23].

The *SLCO1B1* locus encodes a liver-specific transporter of thyroid hormone as well as estrogens which impact liver production of SHBG [24]. *JMJD1C* (10q21.3), also known as *TRIP 8* (thyroid hormone receptor interactor protein 8 [25]), may impact SHBG concentrations via thyroid hormone effects on liver protein production. Thyroid hormone may alter SHBG production through effects on *HNF4*α which is known to regulate *SHBG* transcription [26], [27].

Many of the genes identified are involved in carbohydrate and lipid metabolism and liver function. The *GCKR* locus (2p23.3) encodes a protein that regulates glucokinase activity and has been associated with T2D in several ethnic populations [28], [29], [30], [31]. *GCKR* has been associated with metabolic and inflammatory traits including triglyceride concentrations and other lipid fractions [30], [32], fasting plasma glucose [33], [34], insulin concentrations, uric acid, c-reactive protein (CRP), and non-alcoholic fatty liver disease which are all characteristic of the metabolic syndrome and T2D [28], [35], [36], [37], [38], [39], [40], [41], [42]. The *SLCO1B1* locus (12p12.1) codes for a protein, hepatocyte protein anion-transporting polypeptide 1B1, involved in liver metabolism of both endogenous and exogenous compounds [43]. Consistent with *SLCO1B1's* role in liver metabolism, the same SNP (rs4149056) has been associated with circulating bilirubin concentrations in previous GWAS [44]. *BAIAP2L1* (7q21.3) encodes a protein important in cytoskeleton organization [45] that has been associated with the inflammatory marker CRP in patients with arthritis [46]. *BAIAP2L1* is also known as *IRTKS* (insulin receptor tyrosine kinase substrate) which is involved in insulin receptor signaling [47] and may relate to insulin resistant states including obesity and T2D [48], [49], [50], [51], [52], [53], [54]. We conducted a targeted analysis of *PPARγ*, a gene that influences *SHBG* gene expression in the liver [1], [55] and is associated with T2D [56], [57]. Our analysis identified one significant SNP (rs2920502, p = 9.9×10^−5^) and a second nominally significant SNP (rs13081389, p = 0.01) at *PPARγ*. Some of the identified genes involved in hepatic metabolism of lipids and carbohydrates may be affect SHBG concentrations indirectly through effects on the SHBG transcription regulator *HNF4*α although *HNF4*α itself was not identified in this meta-analyses [27], [58], [59], [60].

The *UGT2B15* locus (4q13.2) was significantly associated with SHBG concentrations in men but not women in this meta-analysis. *UGT2B15* belongs to a family of genes (the UGT2B gene family) that code for enzymes involved in the metabolism of sex hormones through glucuronidation which allows for excretion of sex steroids through the kidney and the gut via bile excretion [61], [62], primary clearance mechanisms for sex steroids [63]. UGT2B15 is involved in the conjugation and inactivation of testosterone [64]. An association between rs293428 in the *UGT2B15* locus and circulating SHBG concentrations in men is supported by a previous study demonstrating that a non-synonymous SNP in *UGT2B15* (rs1902023; D85Y) is associated with serum SHBG concentrations in younger adult men [65]. *UGT2B15* is thought to play a significant role in local tissue inactivation of androgens in androgen dependent prostate cancer [66], [67]. The mechanism behind the influence of genetic variants in *UGT2B15* on SHBG concentrations is unknown, but one may speculate that *UGT2B15* affects the local androgenic environment in selected tissues, which in turn results in regulation of SHBG concentrations.

In addition to *UGT2B15*, three other genes near the identified SNPs are associated with carcinogenesis, particularly in the prostate and breast. *ZBTB10* (8q21.13), has been linked to breast cancer [68]. In breast cancer cell lines *ZBTB10* is suppressed by ROS-microRNA27a thereby enhancing ERα alpha expression and mediating estrogen effects [17]. The *ZNF652* (17q21.32) locus codes for a DNA binding protein thought to act as a tumor suppressor gene in breast cancer [69], [70], [71] that is also co-expressed with the androgen receptor in prostate cancer [72]. *TDGF3*, teratocarcinoma derived growth factor 3, is the only significant region identified on the X chromosome ((Xq22.3). *TDGF3* is a *pseudogene* of *TDGF1* located on chromosome 3p23-p21 that has been associated with testicular germ cell tumors [73].

### Strengths and Limitations

This GWAS meta-analysis incorporated data from approximately 22,000 men and women from 16 epidemiologic cohorts. The overall size of the study yields power but the meta-analysis of data from different epidemiologic studies requires the inclusion of different laboratory methods. The different studies used a variety of assay methodologies to measure serum SHBG concentrations although the vast majority were immunoassays (Tables S1 and S2, Text S1) with similar methodologies. Variation introduced by the use of different SHBG assays would result in loss of statistical power and likely bias toward the null. Additionally, the majority of women were post-menopausal as ascertained by self-report in all studies (Table S1). SHBG concentrations, like testosterone, decline only slightly across the menopause [74] so adjustment for menopause status is not necessary. SHBG may also increase with ovulation and be slightly higher in the luteal versus the follicular phase of the menstrual cycle in premenopausal women, but most studies did not collect data on menstrual phase at the time of SHBG measurement so adjustment for menstrual phase was not possible [75]. Finally, individuals were not excluded based on health status, therefore some individuals with chronic conditions that may affect hepatic production of or clearance of proteins including SHBG such as liver disease, renal disease, or severe malnutrition, may have been included in this analysis.

### Conclusion

SHBG synthesis in the liver is known to be affected directly or indirectly by estrogens, androgens and thyroid hormones and has been observed to be inversely associated with the higher insulin concentrations characteristic of insulin resistant states such as T2D [1], [6]. In summary, the results of this GWAS reflect these influences. Three regions map to proteins related to hepatic function (12p12.1-*SLCO1B1* [*76*], 2p23.3-*GCKR* [*77*] and 10q21.3-*JMJD1C* [*77*]). In addition, 2p23.3-*GCKR* and 7q21.3-*BAIAP2L1* [alias insulin receptor tyrosine kinase substrate (*IRTKS*)] are involved in susceptibility to T2D [48] and insulin signaling [47], respectively. Two signals also mapped to loci involved in thyroid hormone regulation (10q21.3-*JMJD1C* and 12p12.1-*SLCO1B1*). One signal mapped to the receptor for luteinizing hormone 2p16.3-*LHCGR* [*20*], the hormone that stimulates testosterone production. Five regions mapped to genes previously implicated in androgen and estrogen signaling (1p13.3-*PRMT6* [*18*], 8q21.13-*ZBTB10* [*17*], 12p12.1-*SLCO1B1* [*76*], 15q26.2-*NR2F2* [*78*], 4q13.2-*UGT2B15* [*63*]).

We have combined a conventional GWAS approach with detailed additional analyses, including sex stratification, conditional analysis and imputation from 1000 Genomes. Our results demonstrate that these approaches can lead to an appreciable gain in heritable variance explained. It does however highlight the complexity of elucidating individual variant causality through statistical approaches. In addition to the extensive allelic heterogeneity at the *SHBG* locus, our data identify loci with a role in sex steroid hormone metabolism, which may help elucidate the role of sex steroid hormones in disease, particularly T2D and hormone-sensitive cancers.

## Methods

We performed a genome wide association study (GWAS) meta-analysis of 21,791 individuals (Table S1: 9,390 women, 12,401 men) from ten observational studies. Data from an additional six studies totaling 7,046 individuals (Table S2: 4,509 women; 2,537 men) were used for validation. The proportion of variance explained was estimated in an independent study (InCHIANTI, n = 1,129). The individual study protocols were approved by their respective institution's ethics committee/institutional review board and all participants provided informed consent prior to participation. Individuals known to be taking hormonal contraceptives or hormone replacement therapy at time of SHBG measurement were excluded from analysis. Age, sex and body mass index (BMI) were included as covariates. After applying standard quality control measures, imputed genotypes were available for approximately 2.5 M SNPs. See Figure 2 for an overview of the analytic plan and the Text S1 for further information for individual studies included in this meta-analysis.

### GWAS Conditional Meta-Analysis Steps

#### Conditional analysis #1

The initial starting point for the conditional analysis was the four *SHBG* locus SNPs that all showed low Hapmap LD (r^2^<0.05) with each other: rs12150660 (lead SNP Table 1), rs6258 p = 2.7×10^−46^, rs1625895 p = 1.2×10^−14^ and rs3853894 p = 2.5×10^−11^. Each cohort fitted a single regression model, fitting SHBG concentrations against these four genome-wide significant SHBG locus SNPs (rs12150660, rs6258, rs1625895 and rs3853894), in addition to age, sex and BMI. After meta-analyzing the results from all cohorts, three of the SNPs retained genome wide significance when regressed against each other, with the fourth SNP narrowly missing that threshold (rs3853894, p = 4.1×10^−6^).

#### Conditional GWAS #1 (Table 1, conditional analysis)

We next performed a conditional GWAS meta-analysis, where each study included, as additional covariates to the original analysis plan, the ten genome-wide significant autosomal SNPs (the eight ‘Main’ signals from Table 1 and the two unique *SHBG* locus signals described above in addition to the lead SNP rs12150660: rs6258 and rs1625895). Three additional signals (independence based on HapMap r^2^<0.05) at the *SHBG* locus reached genome-wide significance (rs1641537 p = 7.8×10^−32^, rs6259 p = 1.5×10^−12^ and rs10432029 p = 3×10^−8^), giving a total of six independent signals in this gene region. In addition, two novel signals reached genome-wide significance in the conditional analysis, at 7q21.3 (rs3779195 p = 1×10^−8^) and 2p16.3 (rs10454142 p = 3×10^−8^). After replication, only rs3779195 at the *BAIAP2L1* locus retained genome-wide significance.

#### Conditional analysis #2 (Table 2, full model)

Given the six signals observed at the *SHBG* locus (three through conditional analysis #1 rs12150660, rs6258, rs1625895, three through LD estimates from conditional GWAS #1: rs1641537, rs6259, rs10432029), we sought to confirm which of these six were truly independent by a second round of conditional analysis. All discovery and replication cohorts fitted a single regression model of the six SNPs (SNPs # 1–6, Table 2) against SHBG concentrations, using the same parameters and covariates as conditional analysis #1. Four of the six SNPs (#1–4: rs12150660, rs6258, rs1641537, and rs1625895) retained genome-wide significance when conditioned against each other, with two showing nominal evidence of association (SNP #5 rs6259, p = 0.0001; SNP #6 rs10432029, p = 0.01).

#### Conditional GWAS #2 (Table 2, conditional model)

Finally, we performed a second conditional GWAS analysis, adjusting for the six *SHBG* locus SNPs which had evidence of association from conditional analysis #2. All the discovery cohorts were used in this analysis, in addition to three replication cohorts (total sample size 24,354). This analysis revealed evidence for a further three independent signals at the *SHBG* locus (based on HapMap r^2^<0.01), SNP #7 rs9901675 p = 1.5×10^−7^, SNP #8 rs8077824 p = 4.6×10^−5^, and SNP #9 rs9393218 p = 9.9×10^−6^.

### Sensitivity Analysis—Allelic Heterogeneity at the SHBG Locus

We performed a sensitivity analysis using samples from the 1966 Northern Finland Birth Cohort (NFBC1966) study to further investigate allelic heterogeneity at the *SHBG* locus (Text S1). The conditional meta-analysis showed evidence for up to nine signals at the *SHBG* locus, but it is possible that these signals could be explaining a much smaller number of causal variants in the region. Since 1000 Genomes imputation allows us to assess the genetic variation associated with a phenotype across a much denser set of markers, it increases our power to detect allelic heterogeneity within a region. Therefore, 1000 Genomes imputation was carried out on all the samples in the NFBC1966 study and forward selection was used to identify the set of SNPs that best explain the variation in the SHBG phenotype. 1000 Genomes imputation was carried out using IMPUTE2. The mean genotype probabilities for each SNP were calculated and used in the model selection step. Only SNPs 250 kb upstream and 250 kb downstream from the *SHBG* locus (7283453–7786700 bp) were used in the analysis. All SNPs with MAF <0.1% or an imputation quality score less than 0.4 were excluded from the analysis. In total, 1978 *SHBG* region SNPs measured or imputed in 4467 samples from the NFBC1966 study were used in the sensitivity analysis. Forward selection was implemented in R (version 2.13.0) using the stepAIC package to estimate the Akaikie Information Criterion (AIC), an inclusion parameter. Given the high degree of correlation between the SNPs in this region, we increased the penalty (k) on the number of terms included in the model to 12 (where it is usually two), to minimize possible over fitting. The final model included seven SNPs, adjusted for sex and BMI.

### Pathway Analysis

We examined potential interactions among the proteins encoded by the *SHBG* locus and the proteins encoded by the 11 genes (*ZBT10*, *TDGF1*, *ZNF652*, *PRMT6*, *JMJD1C*, *GCKR*, *BAIAP2L1*, *LHCGR*, *SLCO1B1*, *UGT2B15*, *NR2F2*) closest to the 11 identified SNPs using pathway analysis with Search Tool for the Retrieval of Interacting Genes/Proteins (STRING) Pathways Analysis (www.string-db.org). The interactions explored by STRING include direct (physical) and indirect (functional) associations. We then expanded the analysis to examine protein interactions among the *SHBG* gene and the proteins encoded by 67 genes within 500 kb of the 11 identified SNPs.

### Targeted Candidate Gene Analysis

We conducted targeted analysis of two strong candidate genes, hepatocyte nuclear factor-4α (*HNF4*α) and peroxisome-proliferating receptor γ (*PPARγ*). Statistical significance thresholds were set correcting for the number of SNPs tested in each gene region (±100 kb).

## Supporting Information

Table S1 Characteristics of 21,791 individuals from 10 discovery cohorts included in the meta-analysis.
(DOC)

Table S2 Characteristics of 8,175 individuals from the six cohorts included in the validation analysis (WHI, CARDIA, Prospect-EPIC, MrOs, NHS, YFS) and the independent cohort used to estimate the proportion of genetic variance explained by the indentified SNPs (InChianti).
(DOC)

Table S3 Hapmap (release 22) linkage disequilibrium estimates for the nine *SHBG* gene locus single nucleotide polymorphisms.
(DOC)

Text S1 Supplementary Methods with Specific Cohort Information.
(DOC)
