# Supplementary material for: A Genome-Wide Association Meta-Analysis of Circulating Sex Hormone–Binding Globulin Reveals Multiple Loci Implicated in Sex Steroid Hormone Regulation
Source: PLoS Genet. 2012 Jul 19;8(7):e1002805. doi: 10.1371/journal.pgen.1002805 (PMC3400553; doi:10.1371/journal.pgen.1002805)
Supplement: Table S1 — Characteristics of 21,791 individuals from 10 discovery cohorts included in the meta-analysis. (DOC) [file pgen.1002805.s001.doc]

**Table S1: Characteristics of 21,791 individuals from 10 discovery cohorts included in the meta-analysis.**

|  | **FHS** | **GOOD** | **HABC** | **KORA** | **MESA** | **NFBC1966** | **RS-I** | **SHIP** | **TWINS UK** | **YFS** |
| --- | --- | --- | --- | --- | --- | --- | --- | --- | --- | --- |
|  | **Men (N = 12,401)** | | | | | | | | | |
| **N** | 3154 | 936 | 773 | 875 | 1196 | 2513 | 687 | 1528 | NA | 739 |
| **Age, years** | 49.2 (13.7) | 18.9 (0.6) | 73.9 (2.9) | 61.2 (8.9) | 62.7 (10.2) | 31.2 (0.4) | 69.0 (8.0) | 55.4 (15.4) |  | 37.9 (5.0) |
| **BMI, kg/m2** | 28.3 (4.6) | 22.3 (3.2) | 27.1 (3.7) | 28.3 (4.8) | 28.0 (4.1) | 25.2 (3.6) | 25.7 (3.0) | 28.3 (4.2) |  | 26.8 (4.3) |
| **Current smoking, %** | 16.2 | 8.6 | 5.4 | 16.4 | 11.0 | 48.8 | 30.4 | 27.8 |  | 21.6 |
| **SHBG, nmol/L** | 48.4 (25.4) | 20.4 (7.4) | 52.8 (27.2) | 29.7 (12.2) | 44.7 (18.0) | 33.4 (13.4) | 35.8 (13.2) | 32.9 (15.8) |  | 31.1 (12.0) |
|  | **Women (N = 9,390)** | | | | | | | | | |
| **N** | 2503 | NA | 461 | 772 | 1112 | 1954 | 815 | 1376 | 397 | NA |
| **Age, years** | 49.0 (13.5) |  | 73.8 (2.9) | 60.9 (9.0) | 65.1 (9.1) | 31.2 (0.4) | 71.5 (9.2) | 54.9 (14.9) | 54.4 (10.3) |  |
| **Post-menopause, %** | 37.5 |  | 100.0 | 53.8 | 96.6 | NA | 100.0 | 38.4 | 57.0 |  |
| **BMI, kg/m2** | 26.9 (6.2) |  | 26.4 (4.7) | 28.0 (5.8) | 27.6 (5.7) | 24.4 (4.9) | 26.7 (3.8) | 28.1 (5.6) | 25.9 (4.3) |  |
| **Current smoking, %** | 14.9 |  | 6.9 | 12.7 | 11.0 | 36.1 | 17.9 | 23.0 | 41.6 |  |
| **SHBG, nmol/L** | 81.9 (43.9) |  | 64.9 (36.6) | 36.5 (16.7) | 92.3 (63.0) | 86.0 (94.5) | 45.2 (17.8) | 54.5 (36.8) | 57.1 (25.4) |  |
|  | **Men & women (N = 21,791)** | | | | | | | | | |
| Blood sample | Fasting | Non-fasting | Fasting | Fasting | Fasting | Fasting | Non-fasting | Non-fasting | Fasting | Non-fasting |
| **Assay** | Immunoflourometric Assay (Delfia-Wallac, Turku, Finland) | IRMA | Immulite | CLIA | Immulite | Fluoroimmunoassay | RIA | Immulite 2500 | RIA | Fluoroimmunoassay |
| **Genotyping platform & SNP panel** | Affy 5.0+Affy 50K | Illumina HumanHap 610K | Illumina 1M | Affymetrix 6.0 (1000K) | Affimetrix 6.0 | Illumina HumanCNV-370DUO | Illumina Humanhap 550K | Affy 6.0 | Illumina 300K and 610K | Illumina 670k |
| **Genotyping calling algorithm** | BRLLM | BeadStudio | Illumina BeadStudio | Birdseed2 | Birdseed v2 | Beadstudio | Beadstudio | Birdseed2 | Illluminus | Illuminus |
| **Average call rate** | >97% | >97.5% | 97% | 98.85% | >95% | ≥95% | 99.5% | 98.5% | 95% | 99.8% |
| **Imputation software** | MACH 1.0.15 | MACH 1.0 | MACH | IMPUTE 0.4.2 | IMPUTE 2.1.0 | IMPUTE 2.1.0 | MACH | IMPUTE | IMPUTE | MACH 1.0 |

**Data presented in percentages or mean (standard deviation).**
