# Supplementary material for: A Genome-Wide Association Meta-Analysis of Circulating Sex Hormone–Binding Globulin Reveals Multiple Loci Implicated in Sex Steroid Hormone Regulation
Source: PLoS Genet. 2012 Jul 19;8(7):e1002805. doi: 10.1371/journal.pgen.1002805 (PMC3400553; doi:10.1371/journal.pgen.1002805)
Supplement: Table S2 — Characteristics of 8,175 individuals from the six cohorts included in the validation analysis (WHI, CARDIA, Prospect-EPIC, MrOs, NHS, YFS) and the independent cohort used to estimate the proportion of genetic variance explained by the indentified SNPs (InChianti). (DOC) [file pgen.1002805.s002.doc]

**Table S2: Characteristics of 8,175 individuals from the six cohorts included in the validation analysis (WHI, CARDIA, Prospect-EPIC, MrOs, NHS, YFS) and the independent cohort used to estimate the proportion of genetic variance explained by the indentified SNPs (InCHIANTI).**

|  | **Women’s Health Initiative** | **CARDIA Women’s Study** | **Prospect - EPIC** | **MrOS** | **NHS** | **InCHIANTI** | **YFS** |
| --- | --- | --- | --- | --- | --- | --- | --- |
|  | **Men (N =3,044)** | | | | | | |
| **N** | NA | NA | NA | 2537 | NA | 507 | NA |
| **Age, years** |  |  |  | 75.4 (3.2) |  | 66.5 (15.3) |  |
| **BMI, kg/m2** |  |  |  | 26.3 (3.6) |  | 27 (3.4) |  |
| **Current smoking, %** |  |  |  | 8.5 |  | 25.6 |  |
| **SHBG, nmol/L** |  |  |  | 43.2 (21.9) |  | 99.4 (54.9) |  |
|  | **Women (N = 5,131)** | | | | | | |
| **N** | 1803 | 373 | 875 | NA | 779 | 622 | 679 |
| **Age, years** | 67.1 (6.7) | 42.7 (3.3) | 59.3 (5.7) |  | 61.5 (4.8) | 68.3 (15.0) | 32.2 (4.9) |
| **Post-menopause, %** | 100.0 | 5.9 | 100.0 |  | 100.0 | 74.0 | 0.0 |
| **BMI, kg/m2** | 29.7 (5.8) | 27.6 (7.1) | 26.3 (4.2) |  | 26.4 (4.9) | 27.2 (4.7) | 24.7 (4.8) |
| **Current smoking, %** | 11.1 | 18.8 | 23.7 |  | 29.3 | 25.6 | 18.7 |
| **SHBG, nmol/L** | 45.2 (24.0) | 28.3 (11.4) | 23.7 (17.4) |  | 48.0 (1.7) | 138.4 (96.6) | 56.5 (29.1) |
|  | **Men & women (N = 8,175)** | | | | | | |
| **Serum sample** | Fasting | Non-fasting | Non-fasting | Non-fasting | Fasting | Fasting | Non-fasting |
| **Assay** | CLIA | Equilibrium Dialysis | RIA | IRMA | RIA | RIA | Fluoroimmunoassay |
| **Genotyping platform & SNP panel** | Human Omni 1M Quad v1_B SNP | Affymetrix 6.0 | KASPar | KASPar/Taqman | Illumina 550k | Illumina 550k | Illumina 670k |
| **Genotyping calling algorithm** | BeadStudio | Birdseed | Klustercaller | NA | Illuminus | BeadStudio | Illuminus |
| **Average call rate** | 99.96% | ≥98% | 0.98 | > 95% | 99.8% | 98% | 99.8% |
| **Imputation software** | BEAGLE | BEAGLE | NA | NA | MACH 1.0 | MACH | MACH 1.0 |

**Data presented in percentages or mean (standard deviation).**
