# Supplementary material for: A Genome-Wide Association Meta-Analysis of Circulating Sex Hormone–Binding Globulin Reveals Multiple Loci Implicated in Sex Steroid Hormone Regulation
Source: PLoS Genet. 2012 Jul 19;8(7):e1002805. doi: 10.1371/journal.pgen.1002805 (PMC3400553; doi:10.1371/journal.pgen.1002805)
Supplement: Table S3 — Hapmap (release 22) linkage disequilibrium estimates for the nine SHBG gene locus single nucleotide polymorphisms. (DOC) [file pgen.1002805.s003.doc]

**Table S3: HapMap (release 22) linkage disequilibrium estimates for the nine *SHBG* gene locus single nucleotide polymorphisms.**

| **R^2^** | **MAF** | 0.2 | 0.017 | 0.092 | 0.133 | 0.258 | 0.083 | 0.142 | 0.008 | 0.158 |
| --- | --- | --- | --- | --- | --- | --- | --- | --- | --- | --- |
| **MAF** | **SNP** | **rs12150660** | **rs6258** | **rs1625895** | **rs1641537** | **rs9303218** | **rs9901675** | **rs6259** | **rs8077824** | **rs10432029** |
| 0.2 | **rs12150660** | 1 | 0.004 | 0 | 0.038 | 0.04 | 0.023 | 0.041 | 0.002 | 0.047 |
| 0.017 | **rs6258** | 0.004 | 1 | 0.002 | 0.003 | 0.049 | 0.002 | 0.003 | 0 | 0.003 |
| 0.092 | **rs1625895** | 0 | 0.002 | 1 | 0.002 | 0.015 | 0.009 | 0.017 | 0.001 | 0.019 |
| 0.133 | **rs1641537** | 0.038 | 0.003 | 0.002 | 1 | 0.054 | 0.014 | 0.025 | 0.001 | 0.054 |
| 0.258 | **rs9303218** | 0.04 | 0.049 | 0.015 | 0.054 | 1 | 0.012 | 0.402 | 0.003 | 0.066 |
| 0.083 | **rs9901675** | 0.023 | 0.002 | 0.009 | 0.014 | 0.012 | 1 | 0.015 | 0.001 | 0.133 |
| 0.142 | **rs6259** | 0.041 | 0.003 | 0.017 | 0.025 | 0.402 | 0.015 | 1 | 0.001 | 0.031 |
| 0.008 | **rs8077824** | 0.002 | 0 | 0.001 | 0.001 | 0.003 | 0.001 | 0.001 | 1 | 0.002 |
| 0.158 | **rs10432029** | 0.047 | 0.003 | 0.019 | 0.054 | 0.066 | 0.133 | 0.031 | 0.002 | 1 |
|  | | | | | | | | | | |
| **D'** | **MAF** | 0.2 | 0.017 | 0.092 | 0.133 | 0.258 | 0.083 | 0.142 | 0.008 | 0.158 |
| **MAF** | **SNP** | **rs12150660** | **rs6258** | **rs1625895** | **rs1641537** | **rs9303218** | **rs9901675** | **rs6259** | **rs8077824** | **rs10432029** |
| 0.2 | **rs12150660** | 1 | 1 | 0.091 | 1 | 0.677 | 1 | 1 | 1 | 1 |
| 0.017 | **rs6258** | 1 | 1 | 1 | 1 | 1 | 1 | 1 | 1 | 1 |
| 0.092 | **rs1625895** | 0.091 | 1 | 1 | 0.056 | 0.648 | 1 | 1 | 1 | 1 |
| 0.133 | **rs1641537** | 1 | 1 | 0.056 | 1 | 1 | 1 | 1 | 1 | 0.257 |
| 0.258 | **rs9303218** | 0.677 | 1 | 0.648 | 1 | 1 | 0.613 | 0.921 | 1 | 1 |
| 0.083 | **rs9901675** | 1 | 1 | 1 | 1 | 0.613 | 1 | 1 | 1 | 0.525 |
| 0.142 | **rs6259** | 1 | 1 | 1 | 1 | 0.921 |  | 1 | 1 | 1 |
| 0.008 | **rs8077824** | 1 | 1 | 1 | 1 | 1 |  | 1 | 1 | 1 |
| 0.158 | **rs10432029** | 1 | 1 | 1 | 0.257 | 1 | 0.525 | 1 | 1 | 1 |
