# Supplementary material for: A Genome-Wide Association Meta-Analysis of Circulating Sex Hormone–Binding Globulin Reveals Multiple Loci Implicated in Sex Steroid Hormone Regulation
Source: PLoS Genet. 2012 Jul 19;8(7):e1002805. doi: 10.1371/journal.pgen.1002805 (PMC3400553; doi:10.1371/journal.pgen.1002805)
Supplement: Text S1 — Supplementary Methods with Specific Cohort Information. (DOC) [file pgen.1002805.s004.doc]

**Text S1. Supplementary Methods and Cohort Specific Information**

**SUPPLEMENTARY METHODS**

**Sensitivity Analysis**

**Model selection in NFBC1966 study using 1000 genomes imputed data**

We performed a sensitivity analysis using samples from the Northern Finland Birth Cohort 1966 (NFBC1966) study to further investigate allelic heterogeneity at the SHBG locus. The conditional meta-analysis showed evidence for up to 9 signals at the SHBG locus, but it is possible that these signals could be explaining a much smaller number of causal variants in the region. Since 1000 Genomes imputation allows us to assess the genetic variation associated with a phenotype across a much denser set of markers, it increases our power to detect allelic heterogeneity within a region. Therefore, 1000 Genomes imputation was carried out on all the samples in the NFBC1966 study and forward selection was used to identify the set of SNPs that best explain the variation in the SHBG phenotype.

**1000 Genomes Imputation**

1000 Genomes imputation was carried out using IMPUTE2. The mean genotype probabilities for each SNP were calculated and used in the model selection step. Only SNPs 250kb upstream and 250kb downstream from the SHBG locus (7283453 – 7786700 bp) were used in the analysis. All SNPs with MAF < 0.1% or an imputation quality score less than 0.4 were excluded from the analysis. In total, 1978 SNPs were used in the sensitivity analysis.

**Forward Selection**

Forward selection was implemented in R (version 2.13.0) using the stepAIC package. This analysis was done across 1978 SNPs in the SHBG region using 4467 samples from the NFBC66 study. An Akaikie Information Criterion (AIC) was used as an inclusion parameter, but given the high degree of correlation between the SNPs in this region, we increased the penalty (k) on the number of terms included in the model to 12 (where it is usually 2), to minimize possible overfitting. The final model included 7 SNPs, adjusted for BMI and sex. The results of the model selection are shown below and compared with the univariate results obtained by regressing each of the 7 SNPs, adjusted for BMI and sex, against the phenotype.

Table showing the β coefficients (B), standard error (SE) and P-values for each SNP in the multivariate model identified by model selection. For the univariate model, each SNP (in italics), adjusted for sex and BMI, was regressed against the natural logarithm of SHBG levels with the same parameters are shown for comparison between models.

|  | **Multivariate Model** | | | **Univariate Model** | | |
| --- | --- | --- | --- | --- | --- | --- |
| **Variable** | **B** | **SE** | **P-value** | **B** | **SE** | **P-value** |
| Sex | 0.691 | 0.015 | < 2e-16 |  |  |  |
| BMI | -0.049 | 0.002 | < 2e-16 |  |  |  |
| *17-7531965* | 0.094 | 0.012 | 8.39E-16 | 0.093 | 0.011 | 2.60E-17 |
| *rs6259* | 0.112 | 0.020 | 2.14E-08 | 0.067 | 0.019 | 4.83E-04 |
| *17-7359510* | 0.755 | 0.121 | 4.97E-10 | 0.797 | 0.123 | 1.20E-10 |
| *17-7388716* | -0.412 | 0.067 | 1.08E-09 | -0.489 | 0.068 | 8.73E-13 |
| *17-7713738* | 0.484 | 0.127 | 1.41E-04 | 0.486 | 0.130 | 1.85E-04 |
| *17-7533461* | 0.210 | 0.055 | 1.40E-04 | 0.244 | 0.056 | 1.37E-05 |
| *17-7537135* | -0.149 | 0.043 | 5.11E-04 | -0.225 | 0.043 | 2.00E-07 |

The table shows that the B-coefficients and P-values differ between the univariate model and the multivariate model for a portion of the SNPs, which could suggest some correlation between SNPs (i.e. haplotype effects). However, the important outcome of this analysis is that stringent model selection parameters suggest that there are multiple signals in this region and, based on the current available data, these signals are all important in explaining and quantifying the variance explained by this locus. Considering only one SNP in this region would underestimate the variance explained by the SHBG locus.

**A Note on Forward Selection**

Forward selection includes or excludes variables from a model based on a bias/variance trade-off. Each additional parameter included in the model, decreases the overall bias, but simultaneously increases the variance of the model, and therefore model selection looks for the optimal balance between these two parameters to find the model that best fits the data at hand. In practical terms, this means that any variable that is correlated (non-independent) with a variable already in the model will be dropped from the analysis. However, in cases where two SNPs are tagging an un-typed causal SNP with a strong effect on the phenotype, model selection may not necessarily include only one of these SNPs in the final model. Instead, since both SNPs are important in quantifying the full amount of variance explained by the un-typed SNP, the combination of these SNPs may well improve the overall model fit and both will be included in the final model as a result. Thus, forward selection cannot explicitly tease apart independent signals, but rather, identifies the smallest set of SNPs, that together, explain the largest portion of the phenotypic variance. By using 1000 Genomes data, we hope to minimize the chance of two variants being included in the model to explain another unseen variant of higher effect size.

**COHORT SPECIFIC INFORMATION**

1. **Framingham Heart Study (FHS)**

The FHS was initiated in 1948 to examine the determinants of cardiovascular disease and its risk factors (http://www.framinghamheartstudy.org/). The Original Cohort comprised 5,209 men and women, aged 28-62 years at enrollment who have undergone routine biennial examinations [[1]](#_ENREF_82). In 1971, 5,124 Offspring of the Original Cohort participants and Offspring spouses, aged 5 to 70 years, were enrolled into the Framingham Offspring Study and have been examined approximately every 4 to 8 years [2,3]. In the 1990s, DNA was obtained for genetic studies from surviving Original cohort and Offspring participants. From 2002 to 2005, 4,095 men and women aged 20 and older with at least one parent in the Offspring cohort were enrolled in the Third Generation Cohort and DNA was obtained for genetic studies at the time of the first examination [[4]](#_ENREF_85). Routine examinations for all FHS cohorts included a standardized physician administered medical history interview and physical examination, direct measurement of cardiovascular risk factors, laboratory assessment, and various questionnaires and noninvasive cardiovascular tests specific to the given examination cycle. Fasting serum samples from Offspring examination 7 (1998 to 2001) and Generation 3 examination 1 (2002-2005) were used to measure SHBG. FHS examinations were approved by the Institutional Review Board of the Boston University Medical Center and all participants provided written informed consent.

**Genotyping Information**

In 2007, genotyping was conducted through the FHS SHARe (SNP Health Association Resource) project on all Original Cohort, Offspring, and Gen 3 participants with DNA using the Affymetrix 500K mapping array in addition to the Affymetrix 50K supplemental array (http://www.ncbi.nlm.nih.gov/projects/gap/cgi-bin/study.cgi?study_id=phs000007). Sample level exclusions included a participant call rate <97%, a per subject heterozygosity ±5 standard deviations away from the mean, or a per subject large Mendelian error rate. Principal components analysis was used to infer axes of variation using a subset of 425,173 SNPs with MAF ≥0.01, HWE p ≥10^-8^, and call rate ≥0.95. SNP weights for 10 principal components (PCs) were inferred using a maximal set of independent individuals. None of the PCs were associated with SHBG, and therefore none were included as covariates in the SNP association analyses. MACH (version 1.0.15) was used to impute all autosomal SNPs on HapMap, using the publicly available phased haplotypes from HapMap (release 22, build 36, CEU population) as a reference panel [[5]](#_ENREF_86). From a total of 534,982 genotyped autosomal SNPs (Affymetrix 500K and 50K combined), we used 378,163 SNPs that were present on HapMap and that passed quality control measures in the FHS sample including MAF ≥0.01, HWE p>10^-6^, callrate >0.97, mishap test of non-random missingness p>10^-9^, and ≤100 Mendelian errors. IMPUTE v0.5 was used to impute X chromosome SNPs. From a total number of 10,886 Chromosome X genotyped SNPs, 7,795 SNPs that met quality control criteria were used in the IMPUTE program.

**SHBG Assay**

SHBG was measured in fasting, serum samples, with a two-site directed immunofluorometric assay with sensitivity 0.5nM and <0.1% cross-reactivity with other circulating proteins (Delfia-Wallac, Inc., Turku, Finland). The CVs were 8.3%, 7.9%, and 10.9% for low, mid, and high pools, respectively.

**Acknowledgements**

The phenotype-genotype association analyses were funded through grants from the NIA R21AG032598 (JMM, KLL), R01HL094755 (ADC, VSR, SB) and R01AG31206 (VSR, SB), R01 AR/AG 41398 (DPK). “This research was conducted in part using data and resources from the Framingham Heart Study of the National Heart Lung and Blood Institute of the National Institutes of Health and Boston University School of Medicine. The analyses reflect intellectual input and resource development from the Framingham Heart Study investigators participating in the SNP Health Association Resource (SHARe) project. This work was partially supported by the National Heart, Lung and Blood Institute's Framingham Heart Study (Contract No. N01-HC-25195) and its contract with Affymetrix, Inc for genotyping services (Contract No. N02-HL-6-4278). A portion of this research utilized the Linux Cluster for Genetic Analysis (LinGA-II) funded by the Robert Dawson Evans Endowment of the Department of Medicine at Boston University School of Medicine and Boston Medical Center.

**Disclosures**

None

1. **Gothenburg Osteoporosis and Obesity Determinants (GOOD) Study**

The GOOD study was initiated to determine both environmental and genetic factors involved in the regulation of bone and fat mass [[6]](#_ENREF_87). Male study subjects were randomly identified in the greater Gothenburg area in Sweden using national population registers, contacted by telephone, and invited to participate. To be enrolled in the GOOD study, subjects had to be between 18 and 20 years of age. There were no other exclusion criteria, and 49% of the study candidates agreed to participate (n = 1,068). The study was approved by the ethics committee at the University of Gothenburg. Written and oral informed consent was obtained from all study participants.

**Genotyping Information**

Genotyping was performed using the Illumina HumanHap610 Quad arrays at the Genetic Laboratory, Department of Internal Medicine, Erasmus Medical Center, Rotterdam, the Netherlands. Genotypes were called using the BeadStudio calling algorithm. Genotypes from 938 individuals passed the sample quality control criteria [exclusion criteria: sample call rate < 97.5%, gender discrepancy with genetic data from X-linked markers, excess autosomal heterozygosity > 0.33 ~ FDR < 0.1%, duplicates and/or first degree relatives identified using IBS probabilities (> 97%), ethnic outliers (3 SD away from the population mean) using multi-dimensional scaling analysis with four principal components]. Across 22 duplicate samples, genotype concordance exceeded 99.9%. Genotypes were imputed for all polymorphic SNPs (521,160 with MAF ≥ 1%, SNP call rate ≥ 98% and HWE p value ≥ 10^-6^) using the MACH software, based upon phased autosomal chromosomes of the HapMap CEU Phase II panel (release 22, build 36), orientated on the positive strand. We carried out association testing for imputed SNPs using a linear regression framework under an additive (per allele) genetic model as implemented in MACH2QTL [[7]](#_ENREF_88). Uncertainty in genotype prediction was accounted for by utilizing the dosage information from MACH.

**SHBG Assay**

Serum SHBG was measured using immunoradiometric assay (Orion Diagnostics, Espoo, Finland) with a limit of detection of 1.3 nmol/liter, intraassay CV of 3%, and interassay CV of 7%.

**Acknowledgements**

Financial support was received from the Swedish Research Council (K2010-54X-09894-19-3, 2006-3832 and K2010-52X-20229-05-3), the Swedish Foundation for Strategic Research, the ALF/LUA research grant in Gothenburg, the Lundberg Foundation, the Torsten and Ragnar Söderberg's Foundation, Petrus and Augusta Hedlunds Foundation, the Västra Götaland Foundation, the Göteborg Medical Society, the Novo Nordisk foundation, and the European Commission grant HEALTH-F2-2008-201865-GEFOS. We would like to acknowledge Maria Nethander at the genomics core facility at University of Gothenburg for statistical analyses. We would also like to thank Dr. Tobias A. Knoch, Luc V. de Zeeuw, Anis Abuseiris, and Rob de Graaf as well as their institutions the Erasmus Computing Grid, Rotterdam, The Netherlands, and especially the national German MediGRID and Services@MediGRID part of the German D-Grid, both funded by the German Bundesministerium fuer Forschung und Technology under grants #01 AK 803 A-H and # 01 IG 07015 G for access to their grid resources. We would also like to thank Karol Estrada, Department of Internal Medicine, Erasmus MC, Rotterdam, the Netherlands for advice regarding the grid resources.

**Disclosures**

None

1. **Health, Aging, and Body Composition (Health ABC) Study**

The goal of the Health ABC Study was to understand how change in body composition and weight-related health conditions might influence incident functional limitation. The cohort of 3,075 healthy European-American and African-American men and women was recruited from Medicare beneficiaries in Memphis, TN and Pittsburgh, PA, USA between 1997 and 1998. At baseline, participants were between 70 and 79 years of age, 46% percent of the women were African-American, as were 37% of the men. Participants at baseline were free of functional limitation, defined as inability to walk up a flight of stairs and/or to walk a quarter of a mile without difficulty. The Health ABC study has been approved by the institutional review boards of the University of Pittsburgh, the University of Tennessee, Memphis, and the University of California, San Francisco.

**Genotyping Information**

Genomic DNA was extracted from buffy coat collected using PUREGENE® DNA Purification Kit during the baseline exam. In 2009, genotyping was performed by the Center for Inherited Disease Research (CIDR) using the Illumina Human1M-Duo BeadChip system. Samples were excluded from the dataset for the reasons of sample failure, genotypic sex mismatch, and first degree relative of an included individual based on genotype data. Genotyping was successful in 2,802 individuals (1,663 Caucasians and 1,139 African Americans). Genotypes were available on 914263 high quality SNPs before imputation.

**SHBG Assay**

Serum SHBG was determined using an automated continuous, chemiluminescent immunoassay system (IMMULITE, Diagnostics Products Corporation, Los Angeles). The IMMULITE SHBG assay has a sensitivity of 0.20 nmol/L with a calibration range of 2-180 nmol/L. Measurements of higher concentrations were obtained by dilution of the original sample, when there was sufficient remaining sample. The inter-assay coefficients of variation calculated using 5% blind duplicate samples for the SHBG.

**Acknowledgements**

This Health ABC Study was supported by NIA contracts N01AG62101, N01AG62103, and N01AG62106. The genome-wide association study was funded by NIA grant 1R01AG032098-01A1 to Wake Forest University Health Sciences and genotyping services were provided by the Center for Inherited Disease Research (CIDR). CIDR is fully funded through a federal contract from the National Institutes of Health to The Johns Hopkins University, contract number HHSN268200782096C. This research was also supported in part by the Intramural Research Program of the National Institute on Aging, NIH, Bethesda, MD.

**Disclosures**

None

1. **Invecchiare in Chianti (InCHIANTI)**

The InCHIANTI study is a population-based epidemiological study aimed at evaluating factors that influence mobility in the older population living in the Chianti region of Tuscany, Italy. Details of the study have been previously reported [8]. Briefly, 1616 residents were selected from the population registry of Greve in Chianti (a rural area; 11,709 residents with 19.3% of the population greater than 65 years of age) and Bagno a Ripoli (Antella village near Florence; 4704 inhabitants, with 20.3% greater than 65 years of age). The participation rate was 90% (n= 1453) and participants ranged between 21–102 years of age. The study protocol was approved by the Italian National Institute of Research and Care of Aging Institutional Review. There were 85 parent-offspring pairs, 6 sib-pairs and 2 half-sibling pairs documented [9]. We investigated any further familial relationships using IBD of 10,000 random SNPs using RELPAIR and uncovered 1 parentoffspring, 79 siblings and 13 half-siblings. We utilized the correct family structure inferred from genetic data for all analyses.

**Genotyping Information**

Genome-wide genotyping was performed using the Illumina Infinium HumanHap550 genotyping chip (ver1 and ver3 chips were used), using 750ng of genomic DNA extracted from whole blood. Chips were scanned on Illumina BeadStation scanners and data analyzed in BeadStudio (version 3; Illumina). We only used DNA samples for which >98% of all SNPs were scored, that were called in >98% of samples and had minor allele frequencies in our sample of >1%. SNPs deviating appreciably from the expected population distribution (Hardy Weinberg Equilibrium p<1x10^-4^) were also excluded from the analyses. We imputed ~2.5 million HapMap SNPs using MACH Imputation quality was determined by the r2hat metric. Analysis was performed using linear regression allele dosage in MACH. We corrected for any over inflation of statistics due to relatedness or residual population admixture by using an inflation factor, generated using EIGENSTRAT.

**SHBG Assay**

Serum SHBG was measured using immunoradiometric assay (Diagnostic Products, Los Angeles, CA, USA) with an MDC of 3.00 nmol/L, and inter- and intraassay CV concentrations for three different concentrations were 3.7, 1.1, and 3.4% and 11.5, 10.3, and 8.7%, respectively.

**Acknowledgements**

The InCHIANTI study baseline (1998-2000) was supported as a “targeted project” (ICS110.1/RF97.71) by the Italian Ministry of Health and in part by the U.S. National Institute on Aging (Contracts: 263 MD 9164 and 263 MD 821336); the InCHIANTI Follow-up 1 (2001-2003) was funded by the U.S. National Institute on Aging (Contracts: N.1-AG-1-1 and N.1-AG-1-2111); the InCHIANTI Follow-ups 2 and 3 studies (2004-2010) were financed by the U.S. National Institute on Aging (Contract: N01-AG-5-0002); supported in part by the Intramural research program of the National Institute on Aging, National Institutes of Health, Baltimore, Maryland. JRBP is a Sir Henry Wellcome Postdoctoral Research Fellow (092447/Z/10/Z).

**Disclosures**

None

1. **Cooperative Health Research in the Region of Augsburg (KORA)**

The KORA study is a series of independent population-based epidemiological surveys and follow-up studies of participants living in the region of Augsburg, Southern Germany. All participants are residents of German nationality identified through the registration and informed consent has been given by all participants [[10]](#_ENREF_89). The studies have been approved by the local ethics committee. The present study includes data of the follow-up study KORA F4 (2006-2008) of the KORA S4 survey (1999/2000). For genotyping, we included 1,814 randomly selected participants of KORA F4. Valid data were available in 1,647 individuals.

**Genotyping Information**

The KORA F4 samples were genotyped with the Affymetrix Human SNP Array 6.0. Hybridization of genomic DNA was done in accordance with the manufacturer’s standard recommendations. Genotypes were determined using Birdseed2 clustering algorithm. For quality control purposes, we applied a positive control and a negative control DNA every 96 samples. On chip level only subjects with overall genotyping efficiencies of at least 93% were included resulting in an average genotyping efficiency of 98% per chip. In addition, the called sex had to agree with the sex in the KORA study database. Imputation of genotypes was performed with the software IMPUTE v0.4.2 based on HapMap II. Genome-wide association tests were performed using SNPTEST v1.1.5 and SNPTEST v2.1.1 and X-chromosome analyses on genotyped SNPs using PLINK.

**SHBG Assay**

In the KORA cohort, SHBG concentrations were determined from fasting **EDTA plasma** samples stored at -80 °C until analysis. An automated chemiluminescent immunometric assays was used (Siemens Immulite 2000 SHBG, ref. L2KSH2, lot 240; Siemens Healthcare Medical Diagnostics, Bad Nauheim, Germany). The assays were performed according to the manufacturer’s recommendations by skilled technical personal in September and October 2009. Commercial control material at two different levels (LSHC 1-2, Siemens Healthcare Medical Diagnostics, Bad Nauheim, Germany) was included in each series. The inter-assay coefficients of variation were 4.0% and 6.6% at SHBG concentrations of 4.7 nmol/L and 76.0 nmol/L, respectively. All samples and controls were assayed in singlicates.

**Acknowledgements**

The KORA research platform was initiated and financed by the Helmholtz Zentrum Munich, German Research Center for Environmental Health, which is funded by the German Federal Ministry of Education and Research (BMBF) and by the State of Bavaria. Part of this work was financed by the German National Genome Research Network (NGFN-2 and NGFNPlus: 01GS0823). Our research was supported within the Munich Center of Health Sciences (MC Health) as part of LMUinnovativ.

**Disclosures**

None

1. **Multi-Ethnic Study of Atherosclerosis (MESA)**

The MESA is a study of the characteristics of subclinical cardiovascular disease (disease detected non-invasively before it has produced clinical signs and symptoms) and the risk factors that predict progression to clinically overt cardiovascular disease or progression of the subclinical disease. MESA researchers study a diverse, population-based sample of 6,814 asymptomatic men and women aged 45-84. Thirty-eight percent of the recruited participants are white, 28 percent African American, 22 percent Hispanic, and 12 percent Asian, predominantly of Chinese descent. Participants were recruited from six field centers across the United States: Wake Forest University, Columbia University, Johns Hopkins University, University of Minnesota, Northwestern University and University of California – Los Angeles [[11]](#_ENREF_90). The data included in this study are from the European-origin subsample at the baseline examination, which was performed between 2000-2002 in six centers. MESA samples were genotyped as a part of the SNP Health Association Resource (SHARe) Project of the National Heart Lung and Blood Institute.

**Genotyping Information**

MESA-SHARe samples were genotyped using the Affymetrix Genome-Wide Human SNP Array 6.0 (Santa Clara, California); self-reported Caucasian participants were included for analysis in the current study. Sample exclusion criteria included heterozygosity > 53% and individual-level genotyping callrate < 95%. Monomorphic SNPs were removed, and there was no filter on HWE or MAF. IMPUTE version 2.1.0 was used to perform imputation for the MESA SHARe Caucasian participants (chromosomes 1-22) using HapMap Phase I and II - CEU as the reference panel (release #24 - NCBI Build 36 (dbSNP b126)).

**SHBG Assay**

Serum SHBG concentrations were measured in the University of Massachusetts Medical Center Sex Hormone Laboratory (Worcester, MA) from fasting blood samples drawn between 7:30 and 10:30 AM, which were stored and transported at -70°C. SHBG was measured by chemiluminescent enzyme immunometric assay using Immulite kits (Diagnostic Products Corporation, Los Angeles, CA). The overall coefficient of variation for SHBG was 9.0%.

**Acknowledgements**

MESA and the MESA SHARe project are conducted and supported by the National Heart, Lung, and Blood Institute (NHLBI) in collaboration with MESA investigators. Support is provided by grants and contracts N01 HC-95159, N01-HC-95160, N01-HC-95161, N01-HC-95162, N01-HC-95163, N01-HC-95164, N01-HC-95165, N01-HC-95166, N01-HC-95167, N01-HC-95168, N01-HC-95169 and RR-024156. Funding support for the sex hormone dataset was provided by grants HL074406 and HL074338. Funding for SHARe genotyping was provided by NHLBI Contract N02-HL-6-4278. Genotyping was performed at the Broad Institute of Harvard and MIT (Boston, Massachusetts, USA) and at Affymetrix (Santa Clara, California, USA) using the Affymetric Genome-Wide Human SNP Array 6.0.

**Disclosures**

None

1. **Northern Finland Birth Cohort 1966 Study (NFBC1966)**

The study population derives from Northern Finland birth cohort 1966 (NFBC1966). In particular, mothers expected to give birth in the two Northern provinces of Oulu and Lapland in 1966 were enrolled in the NFBC1966 study (N=12,058 live births). Primary clinical data collection on parents and the child occurred prenatally and at birth. Data collection on the child continued at ages six months, one year, 14 years (no data from, six months, one year or 14 years are included in this paper), 31 years, with assessment of a wide range of trait measures. Cohort members still living in Northern Finland and those who had moved to the capital area were invited to a clinical examination at age 31 years (N=8,463). The attendees (71% response rate, N=6,007) were adequately representative of the original cohort [12]. Participants provided fasting blood samples for assessment of SHBG concentration that is the focus of the current study, and DNA was also extracted from the blood samples provided at this time.

**Genotyping Information**

Before Infinium genotyping, all samples were typed for a24-SNP panel on the Sequenom MassArray iPLEX platform. This 24-SNP “FingerPrint” panel included a sex-determining assay and 23 high heterozygosity SNPs that are present on the Infinium 370cnvDuo panel, to serve as a sample-tracking check. Any samples replenished with a second aliquot of stock (from Helsinki) were subjected to an independent “FingerPrint”assay, and the concordance of genotypes between the original and new sample was confirmed. DNA samples were plated at 50 ng ul^-1^ for the Il lumina Infinium 370cnvDuo array[13]. The Infinium chemistry was performed using the manufacturer’s protocols

[https://icom.illumina.com/icom/software.ilmn?id=180]. Arrays were hybridized, stained and detected on Illumina scanners. The raw data were processed during Illumina’s “Autocall” stand alone application with the company-supplied cluster definition file, and used for confirmation of the “FingerPrint” SNP genotype concordance and assessment of overall SNP call rates. Samples whose scans did not pass the 95% call rate cutoff were processed a second time. For delivery of genotypes, sample sheets were prepared, all scans that passed QC were loaded into BeadStudio (v3.1), and genotypes were output in Final Report format after application of project specific cluster definitions, generated as described [http://www.illumina.com/downloads/GTDataAnalysis TechNote.pdf]. The Final Reportswere converted to PLINK .lgen style files, and then into standard linkage format .ped files [http://www.illumina.com/downloads/iconnectPLINK Support Illumina WG Arrays.pdf].

Samples were excluded from all analyses for the following reasons: withdrew consent, duplicate samples, gender discrepancies, contaminated samples, related to another individual, no phenotype data. The level of relatedness between all pairs of genotyped individuals was evaluated by estimating the proportion of the genome that was IBD (Purcell et al. 2007). Only one subject from each pair that that shared > 20 % of the genome IBD was used for analysis. We excluded markers from analysis because of lack of HWE (p < 0.0001), call rate <95% and MAF <1%. The physical location of all SNPs is presented using NCBI Build 36. Imputation of genotypes was performed with the software IMPUTE v2 based on HapMap II. Genome-wide association tests were performed using SNPTEST v1.1.5 and X-chromosome analyses on genotyped SNPs using PLINK.

**SHBG Assay**

The blood samples were drawn after overnight fasting in the morning (between 08:00 and 11:00 AM). The samples were stored in -80 C until serum SHBG concentrations were analyzed by fluoroimmunoassay (Delfia-Wallac, Inc. Ltd., Turku, Finland). The intra- and inter-assay coefficients of variation were 1.3% and 5.1%.

**Acknowledgements**

NFBC1966 received financial support from the Academy of Finland (project grants 104781, 120315, 129269, 1114194, Center of Excellence in Complex Disease Genetics and SALVE), University Hospital Oulu, Biocenter, University of Oulu, Finland (75617), the European Commission (EURO-BLCS, Framework 5 award QLG1-CT-2000-01643), NHLBI grant 5R01HL087679-02 through the STAMPEED program (1RL1MH083268-01), NIH/NIMH (5R01MH63706:02), U54 RR020278, ENGAGE project and grant agreement HEALTH-F4-2007-201413, the Medical Research Council, UK (G0500539, G0600705, PrevMetSyn/SALVE) and the Wellcome Trust (project grant GR069224), UK; and the grant DMS-0239427 from the National Science Foundation. The authors would like to thank the Center of Excellence in Common Disease Genetics of the Academy of Finland and Nordic Center of Excellence in Disease Genetics, the Sydantautisaatio (Finnish Foundation of Heart Diseases), the Broad Genotyping Center, D. Mirel, H. Hobbs, J. DeYoung, P. Rantakallio, and M. Koiranen for advice and assistance.

**Disclosures**

None

1. **Rotterdam Study (RS1)**

Subjects were participants of the RS1, a large prospective population-based cohort study of Caucasian subjects aged 55 years and over, living in the Ommoord district of Rotterdam, the Netherlands. The study was designed to investigate the incidence and determinants of chronic disabling diseases in the elderly. Rationale and design have been described previously [[14]](#_ENREF_91). All 10,275 inhabitants aged 55 years and over were invited for baseline examination between August 1990 and June 1993. Of those, 7,983 participated. Among the subjects living independently, the overall response rate was 77 percent for home interview and 71 percent for examination in the research centre, where blood samples were taken. The Rotterdam Study was approved by the medical ethics committee of the Erasmus University Medical School, and written informed consent was obtained from each subject. The current study is based on 687 men and 815 women for whom genome-wide genotype data and plasma SHBG concentrations were available.

**Genotyping Information**

The Rotterdam Study samples were genotyped using the Illumina Infinium HumanHap550 Beadchip. The following sample QC criteria were applied in the GWAS of RS-I, sample call rate ≥97.5%, gender mismatch with typed X-linked markers, evidence for DNA contamination in the samples using the mean of the autosomal heterozygosity >0.33, exclusion of duplicates or first-degree relatives identified using IBS probabilities, exclusion of outliers (three SD away from the population mean) using multi-dimensional scaling (MDS) analysis with four PC. The inclusion/filtering criteria for SNPs were the following: Minor Allele Frequency ≥ 1%, SNP-callrate ≥ 97.5% and HWE-pvalue ≥ 10-6. Genotypes were imputed for all polymorphic SNPs (minor allele frequency >0.01) using the MACH software, based upon phased autosomal chromosomes of the HapMap CEU Phase II panel (release 22, build 36), orientated on the positive strand. Imputation QC metrics from MACH were used for filtering out SNPs with low-quality data. Statistical analysis of imputed data was performed using MACH2QTL implemented in GRIMP [[7]](#_ENREF_88).

**SHBG Assay**

SHBG concentrations were estimated in 12 batches using double-antibody radioimmunoassay kits, purchased from Diagnostic Systems Laboratories (Webster, TX, USA). The results of this assay were compared with the in-house method used ammonium sulphate precipitation [[15]](#_ENREF_92). A correlation coefficient of 0.930 was obtained. Because of the relatively small volumes of serum available, all values reported are single-sample estimations. Intra-assay coefficients of variation, determined on the basis of duplicate results of internal quality control (QC) serum pools with three different levels of each analyte, were below 4%. Since inter-assay variations were relatively large (14%), the results of all batches were normalized by multiplying all concentrations within a batch by a factor, a method which equalized results for the internal quality-control pools. This was considered justified because the results of these pools and the mean results for male and female sera in each assay batch showed very similar patterns [[16]](#_ENREF_93). This procedure reduced the interassay variation to <4%.

**Acknowledgements**

The generation and management of GWAS genotype data for the Rotterdam Study are supported by the Netherlands Organisation of Scientific Research NWO Investments (nr. 175.010.2005.011, 911-03-012). This study is funded by the Research Institute for Diseases in the Elderly (014-93-015; RIDE2), the Netherlands Genomics Initiative (NGI)/Netherlands Organisation for Scientific Research (NWO) project nr. 050-060-810, and funding from the European Commision (HEALTH-F2-2008-201865, GEFOS; HEALTH-F2-2008-35627, TREAT-OA). The Rotterdam Study is funded by Erasmus Medical Center and Erasmus University, Rotterdam, Netherlands Organization for the Health Research and Development (ZonMw), the Research Institute for Diseases in the Elderly (RIDE), the Ministry of Education, Culture and Science, the Ministry for Health, Welfare and Sports, the European Commission (DG XII), and the Municipality of Rotterdam. We thank Pascal Arp, Mila Jhamai, Dr Michael Moorhouse, Marijn Verkerk, and Sander Bervoets for their help in creating the GWAS database. The authors are grateful to the study participants, the staff from the Rotterdam Study and the participating general practitioners and pharmacists. We would like to thank Dr. Tobias A. Knoch, Karol Estrada, Luc V. de Zeeuw, Anis Abuseiris, and Rob de Graaf as well as their institutions the Erasmus Computing Grid, Rotterdam, The Netherlands, and especially the national German MediGRID and Services@MediGRID part of the German D-Grid, both funded by the German Bundesministerium fuer Forschung und Technology under grants #01 AK 803 A-H and # 01 IG 07015 G for access to their grid resources.

**Disclosures**

None

1. **Study of Health in Pomerania (SHIP)**

SHIP is a longitudinal cohort study in West Pomerania, the north-east area of Germany [[17]](#_ENREF_94). From the entire study population of 212,157 inhabitants living in the area, a sample was selected from the population registration offices, where all German inhabitants are registered. Only individuals with German citizenship and main residency in the study area were included. A two-stage cluster sampling method was adopted from the WHO MONICA Project Augsburg, Germany. In a first step, the three cities of the region (with 17,076 to 65,977 inhabitants) and the 12 towns (with 1,516 to 3,044 inhabitants) were selected. Further 17 out of 97 smaller towns (with less than 1,500 inhabitants) were drawn at random. In a second step, from each of the selected communities, subjects were drawn at random, proportional to the population size of each community and stratified by age and gender. Finally, 7,008 subjects aged 20 to 79 years were sampled, with 292 persons of each gender in each of the twelve five-year age strata. In order to minimize drop-outs by migration or death, subjects were selected in two waves. The net sample (without migrated or deceased persons) comprised 6,267 eligible subjects. Selected persons received a maximum of three written invitations. In case of non-response, letters were followed by a phone call or by home visits if contact by phone was not possible. Finally, the SHIP baseline population finally comprised 4,308 (2,116 men) participants (response 68.8%) examined between 1997 and 2001. After five years, between 2002 and 2006, all participants were invited for follow-up visits, in which 3,300 (1,589 men) participants (response 83.6%) were re-examined. All participants gave informed written consent. The study was monitored by a review board of independent scientists. The study protocol is consistent with the principles of the Declaration of Helsinki, as reflected by an a priori approval of the Ethics Committee of the University of Greifswald. Of the 3,300 SHIP follow-up participants we excluded individuals with missing genomic data, serum SHBG measurements, intake of sexual hormones (anatomic-therapeutical-chemical [ATC] code G03), testosterone 5α reductase inhibitors (G04CB), or sexual hormone antagonists (L02B), yielding a final study population of 2,904 individuals (1,376 women).

**Genotyping Information**

The SHIP samples were genotyped using the Affymetrix Human SNP Array 6.0. Hybridization of genomic DNA was done in accordance with the manufacturer’s standard recommendations. The genetic data analysis workflow was created using the Software InforSense. Genetic data were stored using the database Caché (InterSystems). Genotypes were determined using the Birdseed2 clustering algorithm. For quality control purposes, several control samples where added. On the chip level, only subjects with a genotyping rate on QC probesets (QC callrate) of at least 86% were included. All remaining arrays had a sample callrate > 92%. The overall genotyping efficiency of the GWA was 98.55 %. Imputation of genotypes in SHIP was performed with the software IMPUTE v0.5.0 based on HapMap II. Genome-wide association tests were performed using QUICKTEST v0.95 (http://toby.freeshell.org/software/quicktest.shtml). Uncertainties for imputed genotypes were taken into account for association testing.

**SHBG Assay**

The blood samples were drawn from the cubital vein in the supine position between 8:00 AM and 7:00 PM. Serum SHBG concentration was measured from frozen serum aliquots using chemiluminescent enzyme immunoassays on an Immulite 2500 analyzer ([Immulite 2550 SHBG ref. L5KSH, lot 119]; Siemens Healthcare Medical Diagnostics, Bad Nauheim, Germany). One single measurement of SHBG serum concentration was performed. In the SHBG assay, two levels of the manufacturer’s control material, supplied with the reagent (SHBG control [ref. LSHC1 and LSHC2]; Siemens) were included within each series for single determination. During the course of the study, the interassay coefficient of variation was 4.5% with a systematic deviation of +1.1% at the 4.7 nmol/L level and 7.2% with a systematic deviation of +0.3% at the 73 nmol/L level [[18]](#_ENREF_95). All assays were performed according to the manufacturers’ recommendations by skilled technical personnel.

**Acknowledgements**

We thank all staff members and participants of the SHIP study, as well as all of the genotyping staff for generating the SHIP SNP data set. The genetic data analysis workflow was created using the Software InforSense. Genetic data were stored using the database Caché (InterSystems).

**Disclosures**

SHIP is part of the Community Medicine Research net of the University of Greifswald, Germany, which is funded by the Federal Ministry of Education and Research (grants no. 01ZZ9603, 01ZZ0103, and 01ZZ0403), the Ministry of Cultural Affairs as well as the Social Ministry of the Federal State of Mecklenburg-West Pomerania. Genome- wide data have been supported by the Federal Ministry of Education and Research (grant no. 03ZIK012) and a joint grant from Siemens Healthcare, Erlangen, Germany and the Federal State of Mecklenburg West Pomerania. The University of Greifswald is a member of the 'Center of Knowledge Interchange' program of the Siemens AG. This work is also part of the research project Greifswald Approach to Individualized Medicine (GANI_MED). The GANI_MED consortium is funded by the Federal Ministry of Education and Research and the Ministry of Cultural Affairs of the Federal State of Mecklenburg – West Pomerania (03IS2061A). The SHBG reagents used were sponsored by Siemens Healthcare Diagnostics, Eschborn, formerly DPC Biermann GmbH, Bad Nauheim, Germany. Novo Nordisk provided partial grant support for the determination of serum samples and data analysis. RH received honorarium for lectures by Bayer Pharma AG. HW has received research grants from Novo Nordisk and Pfizer for research unrelated to the contents of this manuscript and honorarium for lectures by Bayer Pharma AG.

1. **TWINS UK**

The TwinsUK cohort consisted of a group of twins ascertained to study the heritability and genetics of age-related diseases (www.twinsUK.ac.uk). These unselected twins were recruited from the general population through national media campaigns in the UK and shown to be comparable to age-matched population singletons in terms of disease-related and lifestyle characteristics [19,20]. TwinsUK consists of twins from the adult twin British registry, also shown to be representative of singleton populations and the United Kingdom population [[20]](#_ENREF_97). Serum Sex Hormone Binding Globulin (SHBG) and genome-wide genotype and imputed data were available for 397 samples. Ethics approval was obtained from the Guy’s and St. Thomas’ Hospital Ethics Committee. Written informed consent was obtained from every participant to the study.

**Genotyping Information**

TwinsUK samples were typed with the Infinium 317K, and 610k assay (Illumina, San Diego, USA) at two different centers, the Centre for Inherited Diseases Research (USA) and the Wellcome Trust Sanger Institute. We pooled the normalized intensity data and called genotypes on the basis of the Illluminus algorithm. No calls were assigned if the most likely call was less than a posterior probability of 0.95. Validation of pooling was done by visual inspection of 100 random, shared SNPs for overt batch effects; none were observed. We excluded SNPs that had a low call rate (≤ 95%), Hardy-Weinberg p values < 10−6 and minor allele frequencies < 1%. We, also removed subjects where genotyping failed for >2 % of SNPs. The overall genotyping efficiency of the GWA was 98.7%. Imputation of genotypes was carried out using the software IMPUTE [[5]](#_ENREF_86).

**SHBG Assay**

Serum concentrations of SHBG were measured by automated electrochemiluminescence immunoassay “ECLIA” (Roche Diagnostics, Mannheim, Germany) with a coefficient of variation of less than 10%.

**Acknowledgements**

The study was funded by the Wellcome Trust; European Community’s Seventh Framework Programme (FP7/2007-2013)/grant agreement HEALTH-F2-2008-201865-GEFOS and (FP7/2007-2013), ENGAGE project grant agreement HEALTH-F4-2007-201413 and the FP-5 GenomEUtwin Project (QLG2-CT-2002-01254). The study also receives support from the Dept of Health via the National Institute for Health Research (NIHR) comprehensive Biomedical Research Centre award to Guy's & St Thomas' NHS Foundation Trust in partnership with King's College London. TDS is an NIHR senior Investigator. The project also received support from a Biotechnology and Biological Sciences Research Council (BBSRC) project grant. (G20234) .The authors acknowledge the funding and support of the National Eye Institute via an NIH/CIDR genotyping project (PI: Terri Young). We thank the staff from the Genotyping Facilities at the Wellcome Trust Sanger Institute for sample preparation, Quality Control and Genotyping led by Leena Peltonen and Panos Deloukas; Le Centre National de Génotypage, France, led by Mark Lathrop, for genotyping; Duke University, North Carolina, USA, led by David Goldstein, for genotyping; and the Finnish Institute of Molecular Medicine, Finnish Genome Center, University of Helsinki, led by Aarno Palotie. Genotyping was also performed by CIDR as part of an NEI/NIH project grant.

**Disclosures**

None

1. **The Cardiovascular Risk in Young Finns Study (YFS)**

The YFS is a population-based follow up-study started in 1980 [[21]](#_ENREF_98). The main aim of the YFS is to determine the contribution made by childhood lifestyle, biological and psychological measures to the risk of cardiovascular diseases in adulthood. In 1980, over 3,500 children and adolescents all around Finland participated in the baseline study. The follow-up studies have been conducted mainly with 3-year intervals. The latest 27-year follow-up study was conducted in 2007 (ages 30-45 years) with 2,204 participants.

**Genotyping Information**

Genomic DNA was extracted from peripheral blood leukocytes using a commercially available kit and Qiagen BioRobot M48 Workstation according to the manufacturer’s instructions (Qiagen, Hilden, Germany). Genotyping was done for 2,556 samples using custom build Illumina Human 670k BeadChip at Welcome Trust Sanger Institute. Genotypes were called using Illuminus clustering algorithm. Fifty-six samples failed Sanger genotyping pipeline QC criteria (i.e., duplicated samples, heterozygosity, low call rate, or Sequenom fingerprint discrepancy). From the remaining 2,500 samples one sample failed gender check, three was removed due to low genotyping call rate (< 0.95) and 54 samples for possible relatedness (pi-hat > 0.2) . 11,766 SNPs were excluded based on HWE test (p = 1e-06), 7,746 SNPs failed missingness test (call rate < 0.95) and 34,596 SNPs failed frequency test (MAF < 0.01). After quality control there were 2,442 samples and 546,677 genotyped SNPs available for further analysis. Genotype imputation was performed using MACH 1.0 and HapMap II CEU (release 22) samples as reference. Palindromic A/T and C/G SNPs were removed before imputation. After imputation there were 2,543,887 imputed SNPs available. SNPs with squared correlation between imputed and true genotypes ≥ 0.30 were considered well imputed.

**SHBG Assay**

Serum SHBG concentrations were quantified by commercial time-resolved fluoroimmunoassay kits (Wallace Ltd., Turku, Finland). The interassay coefficients of variation for high, median, and low level controls ranged from 6.6–8.7% for SHBG [[22]](#_ENREF_99).

**Acknowledgements**

The Young Finns Study has been financially supported by the Academy of Finland: grants 134309 (Eye), 126925, 121584, 124282, 129378 (Salve), 117787 (Gendi), and 41071 (Skidi), the Social Insurance Institution of Finland, Kuopio, Tampere and Turku University Hospital Medical Funds (grant 9M048 for 9N035 for TeLeht), Juho Vainio Foundation, Paavo Nurmi Foundation, Finnish Foundation of Cardiovascular Research and Finnish Cultural Foundation, Tampere Tuberculosis Foundation and Emil Aaltonen Foundation (T.L). The expert technical assistance in the statistical analyses by Irina Lisinen and Ville Aalto are gratefully acknowledged.

**Disclosures**

None

1. **Women's Health Initiative (WHI)**

Blood was collected from 27,347 study participants prior to entry into WHI randomized, placebo-controlled clinical trial of hormone therapy in post-menopausal women [[23]](#_ENREF_100). The WHI hormone trial enrolled women aged 50-79 years between 1993-1998, primarily by population-based direct mail strategies, at 40 clinical centers in 24 states and the District of Columbia in the U.S. Details of the study design, recruitment, data collection methods, follow-up, intensive validation and tabulations of baseline data have been published in detail [[23]](#_ENREF_100).

**Genotyping Information**

Genome-wide genotyping was performed as part of the NHGRI-funded Genomics and Randomized Clinical Network (GARNET) at the Broad Institute using the Human Omni 1M Quad v1_B SNP array. Samples were excluded from the dataset for the reasons of genotyping failure, genotypic sex mismatch, and first degree relative of an included individual based on genotype data. A total of 942,499 high-quality SNPs passed QC filters. Genotype imputation was performed at the GARNET Data Coordinating Center (University of Washington) using BEAGLE software and the European continental reference panels selected from the 1000 Genomes Project. Uncertainty in genotype prediction was accounted for by utilizing the dosage information from BEAGLE [[24]](#_ENREF_101). Genotyping data were available on 1,803 Caucasian participants with SHBG measurements.

**SHBG Assay**

Fasting blood samples were collected from each study participant prior to entry into Women's Health Initiative randomized, placebo-controlled clinical trial of hormone therapy in post-menopausal women [[23]](#_ENREF_100). Women who were using hormones at initial contact completed a 3-month washout period prior to the baseline blood collection. Blood was drawn following at least a 12-hour fast, centrifuged at 1300*g* for 10 minutes, and the separated sera were stored in aliquots at −70°C within 2 hours of blood collection. Serum SHBG was assayed at the Reproductive Endocrine Research Laboratory (University of Southern California, Los Angeles, CA, USA), via a solid-phase, two-site chemiluminescent immunoassay using the Immulite analyzer (Siemens Medical Solutions, Malvern, PA, USA) that consists of polystyrene beads coupled to a mouse monoclonal antibody specific for SHBG (Siemens Medical Solutions, USA). The SHGB assay had a sensitivity of 0.2 nmol/L. The intra-assay CVs for the SHBG assay were 2.5% at 21 nmol/L, 2.7% at 63 nmol/L, and 5.3% at 80 nmol/L. The interassay CVs were 5.2% at 21 nmol/L, 5.2% at 63 nmol/L, and 6.6% at 80 nmol/L.

**Acknowledgements**

Genotyping was performed at the Broad Institute (Cambridge, MA) through the NHGRI-funded Genomics and Randomized Clinical Network (U01 HG005152) or GARNET. The WHI program is funded by the National Heart, Lung, and Blood Institute, National Institutes of Health, U.S. Department of Health and Human Services through contracts N01WH22110, 24152, 32100-2, 32105-6, 32108-9, 32111-13, 32115, 32118-32119, 32122, 42107-26, 42129-32, and 44221. The authors thank the WHI investigators and staff for their dedication, and the study participants for making the program possible. A listing of WHI investigators can be found at <http://www.whiscience.org/publications/WHI_investigators_shortlist.pdf>.

**Disclosures**

None

1. **Coronary Artery Risk Development in Young Adults (CARDIA) Women’s Study**

The Coronary Artery Risk Development in Young Adults (CARDIA) study is a multicenter longitudinal cohort study designed to investigate the development of coronary heart disease risk factors in young adults. The initial cohort consisted of 5115 young adult participants, balanced by age (18-24 years and 25-30 years), sex, race (black and white), and educational status (≤ high school graduate, > high school education). Details about eligibility criteria and baseline demographic characteristics have been previously published [[25]](#_ENREF_102). Participants from 4 US cities (Birmingham, Alabama; Chicago, Illinois; Minneapolis, Minnesota; and Oakland, California) were initially examined in 1985-1986. Follow-up examinations were performed 2, 5, 7, 10, and 15years after baseline with retention of the cohort of 91%, 86%, 81%, 79%, and 74%, respectively. The study was approved by the institutional review board at each center and written informed consent was obtained from all participants.

The CARDIA Women’s Study was designed to examine the associations of androgens, polycystic ovaries, and clinical features of the polycystic ovary syndrome with subclinical atherosclerosis. Women eligible for CWS had to have attended the year-15 CARDIA examination, have at least one ovary, and not be pregnant. The CWS examination occurred in 2002-03. The data included in this study are from the European-origin subsample from the CWS examination. Year-15 CARDIA samples were genotyped as a part of the Candidate-gene Association Resource (CARe) of the National Heart Lung and Blood Institute.

**Genotyping Information**

The CARDIA Study samples were genotyped using the Affymetrix Genome-Wide Human SNP Array 6.0 (Santa Clara, California); only participants of European descent were included in the analyses. Genotyping was completed for 1,720 individuals with a sample call rate ≥ 98%. A total of 578,568 SNPs passed quality control (MAF ≥ 2%, call rate ≥ 95%, HWE ≥ 10^-4^) and were used for imputation. Imputation was performed using the BEAGLE algorithm and software [[24]](#_ENREF_101) with the Hapmap2 CEU as the reference population. For this study, complete genotype and phenotype information were available for 373 women.

**SHBG Assay**

Assays were conducted by the Obstetrics and Gynecology Research and Diagnostic Laboratory at the University of Alabama, Birmingham (UAB), a Clinical Laboratory Improvement Amendments (CLIA)-certified reproductive endocrinology laboratory. Serum SHBG was determined using equilibrium dialysis on Sephadex G-25 as described by Pearlman [[26]](#_ENREF_103). This method estimates the amount of testosterone capable of being bound by SHBG. The interassay coefficients of variation (CV) in males and pregnant females were 5.2 and 5.3%, respectively, for the UAB laboratory.

**Acknowledgements**

The CARDIA study is funded by contracts N01-HC-95095, N01-HC-48047, N01-HC-48048, N01-HC-48049, N01-HC-48050, N01-HC-45134, N01-HC-05187, N01-HC-45205, and N01-HC-45204 and the CARDIA Women’s study by R01-HL065611 from the National Heart, Lung, and Blood Institute to the CARDIA investigators. Genotyping of the CARDIA participants was supported by grants U01-HG-004729, U01-HG-004446, and U01-HG-004424 from the National Human Genome Research Institute. Statistical analyses were supported by grants U01-HG-004729 and R01-HL-084099 to MF. MW is supported by the Career Development Award 5-K23-HL087114.

**Disclosures**

None

1. **Prospect - EPIC**

The Prospect-EPIC cohort is one of the two Dutch contributions to the European Prospective Investigation into Cancer and Nutrition (EPIC). The design and rationale of this study has been described previously [[27]](#_ENREF_104). In brief, this cohort consists of 17357 white women living in Utrecht and surroundings, aged 49 – 70 years, who were invited to participate in the study through the national breast cancer screening program between 1993 and 1997. At recruitment, each participant filled out a general questionnaire on lifestyle factors, reproductive (gynecological and obstetric) history, and past and current morbidity as well as a validated (semi)quantitative food frequency questionnaire (FFQ) aimed at capturing the habitual diet during the year preceding enrollment. In addition, pulse rate, blood pressure, and anthropometric measurements were taken, and a 30-ml non-fasting blood sample was donated. Within 24 h, samples of 4 ml serum, 9 ml citrate plasma, 2 ml white blood cells, and 2 ml red blood cells were fractionated into 0.5-ml aliquots, and stored at -196 C. Blood samples were successfully drawn from 97.5% of the women. All women signed a written informed consent before study inclusion. The study complies with the Declaration of Helsinki and was approved by the Institutional Review Board of the University Medical Center Utrecht. Of all Prospect-EPIC participants, a 10% random sample (n = 1,736) was taken, and for the present study, only those who were postmenopausal at recruitment either through natural (no menstrual periods for at least 12 months after spontaneous cessation of their menses) or surgical (hysterectomy, ovariectomy, or a combination) causes, had donated a plasma sample, and did not use hormone replacement therapy or oral contraceptives at the time of the blood donation were included. Genomic DNA was extracted from buffy coat aliquots by KBioscience using their own in-house silica based systems for buffy extractions (<http://www.kbioscience.co.uk/145 purification/purification_intro.html>). For 875 women both DNA and SHBG levels were available.

**Genotyping Information**

KASPar - Genotyping was performed by KBioscience (Herts, U.K.), who designed and used assays based on their proprietary competitive allele-specific PCR (KASPar) method (details of which are available on their website [www.kbioscience.co.uk/](http://www.kbioscience.co.uk/)).

**SHBG Assay**

SHBG was measured in plasma using a commercially available double-antibody radioimmunoassay (RIA) kit (DSL-6300, Diagnostic System Laboratories Inc., Webster, TX, USA). The intra- and inter-assay coefficients of variation were 3.0% and 4.0%, respectively.

**Acknowledgements**

The Prospect-EPIC study was funded by 'Europe against Cancer' Programme of the European Commission (SANCO); Dutch Ministry of Health, Welfare and Sports (VWS); and ZONMw.

**Disclosures**

None

1. **Osteoporotic Fractures in Men (MrOS) Study - Sweden**

The MrOS study is a multicenter, prospective study including 3,014 elderly men in Sweden, Hong Kong (≅2,000), and the United States (≅6,000). The MrOS Sweden cohort consist of three sub-cohorts from three different Swedish cities (n=1,005 in Malmö, n=1,010 in Göteborg, and n=999 in Uppsala). Study subjects (men aged 69–80 years) were randomly identified using national population registers, contacted and asked to participate. To be eligible for the study, the subjects had to be able to walk without assistance, provide self-reported data, and sign an informed consent; there were no other exclusion criteria. The study was approved by the ethics committees at the Universities of Gothenburg, Lund, and Uppsala. Informed consent was obtained from all study participants [[28]](#_ENREF_105).

**Genotyping Information**

Genotyping of all SNPs was carried out on the entire MrOS Sweden cohort for whom DNA was available by KBioscience ([http://www.kbioscience.co.uk](http://www.kbioscience.co.uk/)), who employ a novel form of competitive allele specific PCR (KASPar) and Taqman^TM^ system for genotyping. SNP call rate was ≥95% and all SNPs were in HWE.

**SHBG Assay**

Serum SHBG was measured using immunoradiometric assay (Orion Diagnostics, Espoo, Finland) with a limit of detection of 1.3 nmol/liter, intraassay CV of 3%, and interassay CV of 7%.

**Acknowledgements**

Financial support was received from the Swedish Research Council (2006-3832), the Swedish Foundation for Strategic Research, the ALF/LUA research grant in Gothenburg, the Lundberg Foundation, the Torsten and Ragnar Söderberg's Foundation, Petrus and Augusta Hedlunds Foundation, the Västra Götaland Foundation, the Göteborg Medical Society, the Novo Nordisk foundation and the European Commission grant HEALTH-F2-2008-201865-GEFOS.

**Disclosures**

None

1. **Nurses' Health Study (NHS)**

Details of the NHS cohorts have been described previously [[29]](#_ENREF_106). Briefly, the NHS was initiated in 1976, when 121,700 United States registered nurses between the ages of 30 and 55, residing in 11 larger U.S. states, returned an initial questionnaire reporting medical histories and baseline health-related exposures, including information related to reproductive history (age at menarche, age at first birth, parity, age at menopause etc.), and exposure to exogenous hormones (oral contraception or post-menopausal hormone replacement therapy). Biennial questionnaires with collection of exposure information on risk factors have been collected prospectively, and outcome data with follow-up of reported disease events are collected. From May 1989 through September 1990, we collected blood samples from 32,826 participants in the NHS cohort. Subsequent follow-up has been greater than 99% for this subcohort. Informed consent was obtained from all participants. The study was approved by the Institutional Review Board of the Brigham and Women’s Hospital, Boston, MA, USA.

The NHS nested breast cancer case-control study was derived from the 32,826 women in the blood subcohort who were free of diagnosed breast cancer at blood collection and followed for incidence disease until June 1, 2004. Breast cancer follow-up in the NHS was conducted by personal mailings and searches of the National Death Index. Controls were women not diagnosed with breast cancer during follow-up, and were one-to-one matched to cases based on age at diagnosis, blood collection variables (time of day, season, and year of blood collection, as well as recent (<3 months) use of postmenopausal hormones), ethnicity (all cases and controls are self-reported Caucasians), and menopausal status (all cases were postmenopausal at diagnosis). The 2,287 NHS participants included in the present analysis were from this nested breast cancer case-control study and were self-described Caucasians with genotype data available from the National Cancer Institute’s Cancer Genetic Marker of Susceptibility (CGEMS) project [[30]](#_ENREF_107). There are 1718 women having plasma SHBG levels measured with genotyping data available. Among them 779 women not taking PMH at blood draw were used in this analysis.

**Genotyping Information**

The NHS breast cancer GWA scan used the Illumina Infinium Sentrix HumanHap550 chip. Detailed methods related to the genotyping have been published previously [[30]](#_ENREF_107). Imputation of untyped genotypes was based on HapMap haplotype reference (release 21) using MACH software.

**SHBG Assay**

Plasma SHBG was analyzed in four batches (sent in 1992, 1993, 1996, and 1998). The first 2 batches were assayed at the Longcope Steroid Radioimmunoassay Laboratory at the University of Massachusetts Medical Center (Worcester, MA, USA). The third and fourth of SHBG were assayed by the Massachusetts General Hospital's Reproductive Endocrinology Unit Laboratory (Boston, Massachusetts). Methods used for hormone assays have been described in detail previously [31,32]. SHBG was measured with an immunoradiometric kit from FARMOS Diagnostica (Orion Corp., Turku, Finland) in the Longcope laboratory and with the AxSYM Immunoassay system (Abbott Diagnostics, Chicago, Illinois) in the Massachusetts General Hospital Reproductive Endocrinology Unit laboratory. To allow for assessment of batch-to-batch variation and laboratory precision, we randomly distributed blinded quality control samples (equivalent to 10% of replicates) along with all analytic samples. Within-batch laboratory coefficient of variation was 8% for SHBG.

**Acknowledgements**

The NHS breast cancer GWAS was performed as part of the Cancer Genetic Markers of Susceptibility (CGEMS) initiative of the NCI. We particularly acknowledge the contributions of R. Hoover, A. Hutchinson, K. Jacobs and G. Thomas. The current research is supported by CA87969, CA49449, CA40356, CA128034 and U01-CA98233 from the National Cancer Institute. We acknowledge the study participants in the NHS for their contribution in making this study possible.

**Disclosures**

None

**SUPPLEMENTARY REFERENCES**

1. Dawber TR, Meadors GF, Moore FE, Jr. (1951) Epidemiological approaches to heart disease: the Framingham Study. Am J Public Health Nations Health 41: 279-281.

2. Feinleib M, Kannel WB, Garrison RJ, McNamara PM, Castelli WP (1975) The Framingham Offspring Study. Design and preliminary data. Prev Med 4: 518-525.

3. Kannel WB, Feinleib M, McNamara PM, Garrison RJ, Castelli WP (1979) An investigation of coronary heart disease in families. The Framingham offspring study. Am J Epidemiol 110: 281-290.

4. Splansky GL, Corey D, Yang Q, Atwood LD, Cupples LA, et al. (2007) The Third Generation Cohort of the National Heart, Lung, and Blood Institute's Framingham Heart Study: design, recruitment, and initial examination. Am J Epidemiol 165: 1328-1335.

5. Marchini J, Howie B, Myers S, McVean G, Donnelly P (2007) A new multipoint method for genome-wide association studies by imputation of genotypes. Nat Genet 39: 906-913.

6. Lorentzon M, Swanson C, Andersson N, Mellstrom D, Ohlsson C (2005) Free testosterone is a positive, whereas free estradiol is a negative, predictor of cortical bone size in young Swedish men: the GOOD study. J Bone Miner Res 20: 1334-1341.

7. Estrada K, Abuseiris A, Grosveld FG, Uitterlinden AG, Knoch TA, et al. (2009) GRIMP: a web- and grid-based tool for high-speed analysis of large-scale genome-wide association using imputed data. Bioinformatics 25: 2750-2752.

8. Ferrucci L, Bandinelli S, Benvenuti E, Di Iorio A, Macchi C, et al. (2000) Subsystems contributing to the decline in ability to walk: bridging the gap between epidemiology and geriatric practice in the InCHIANTI study. J Am Geriatr Soc 48: 1618-1625.

9. Melzer D, Perry JR, Hernandez D, Corsi AM, Stevens K, et al. (2008) A genome-wide association study identifies protein quantitative trait loci (pQTLs). PLoS Genet 4: e1000072.

10. Wichmann HE, Gieger C, Illig T (2005) KORA-gen--resource for population genetics, controls and a broad spectrum of disease phenotypes. Gesundheitswesen 67 Suppl 1: S26-30.

11. Bild DE, Bluemke DA, Burke GL, Detrano R, Diez Roux AV, et al. (2002) Multi-ethnic study of atherosclerosis: objectives and design. Am J Epidemiol 156: 871-881.

12. Jarvelin MR, Sovio U, King V, Lauren L, Xu B, et al. (2004) Early life factors and blood pressure at age 31 years in the 1966 northern Finland birth cohort. Hypertension 44: 838-846.

13. Sabatti C, Service SK, Hartikainen AL, Pouta A, Ripatti S, et al. (2009) Genome-wide association analysis of metabolic traits in a birth cohort from a founder population. Nat Genet 41: 35-46.

14. Hofman A, van Duijn CM, Franco OH, Ikram MA, Janssen HL, et al. (2011) The Rotterdam Study: 2012 objectives and design update. Eur J Epidemiol 26: 657-686.

15. de Jong FH, Oishi K, Hayes RB, Bogdanowicz JF, Raatgever JW, et al. (1991) Peripheral hormone levels in controls and patients with prostatic cancer or benign prostatic hyperplasia: results from the Dutch-Japanese case-control study. Cancer Res 51: 3445-3450.

16. Hak AE, Witteman JC, de Jong FH, Geerlings MI, Hofman A, et al. (2002) Low levels of endogenous androgens increase the risk of atherosclerosis in elderly men: the Rotterdam study. J Clin Endocrinol Metab 87: 3632-3639.

17. Völzke H, Alte D, Schmidt CO, Radke D, Lorbeer R, et al. (2011) Cohort profile: the study of health in pomerania. Int J Epidemiol 40: 294-307.

18. Koch B, Glaser S, Schaper C, Krebs A, Nauck M, et al. (2011) Association between serum testosterone and sex hormone-binding globulin and exercise capacity in men: results of the Study of Health in Pomerania (SHIP). J Androl 32: 135-143.

19. Spector TD, Williams FM (2006) The UK Adult Twin Registry (TwinsUK). Twin Res Hum Genet 9: 899-906.

20. Andrew T, Hart DJ, Snieder H, de Lange M, Spector TD, et al. (2001) Are twins and singletons comparable? A study of disease-related and lifestyle characteristics in adult women. Twin Res 4: 464-477.

21. Raitakari OT, Juonala M, Ronnemaa T, Keltikangas-Jarvinen L, Rasanen L, et al. (2008) Cohort profile: the cardiovascular risk in Young Finns Study. Int J Epidemiol 37: 1220-1226.

22. Heikkila R, Aho K, Heliovaara M, Hakama M, Marniemi J, et al. (1999) Serum testosterone and sex hormone-binding globulin concentrations and the risk of prostate carcinoma: a longitudinal study. Cancer 86: 312-315.

23. Stefanick ML, Cochrane BB, Hsia J, Barad DH, Liu JH, et al. (2003) The Women's Health Initiative postmenopausal hormone trials: overview and baseline characteristics of participants. Ann Epidemiol 13: S78-86.

24. Browning BL, Browning SR (2009) A unified approach to genotype imputation and haplotype-phase inference for large data sets of trios and unrelated individuals. Am J Hum Genet 84: 210-223.

25. Friedman GD, Cutter GR, Donahue RP, Hughes GH, Hulley SB, et al. (1988) CARDIA: study design, recruitment, and some characteristics of the examined subjects. J Clin Epidemiol 41: 1105-1116.

26. Pearlman WH (1970) Measurement of testosterone binding sites. Acta Endocrinol Suppl (Copenh) 147: 225-238.

27. Boker LK, van Noord PA, van der Schouw YT, Koot NV, Bueno de Mesquita HB, et al. (2001) Prospect-EPIC Utrecht: study design and characteristics of the cohort population. European Prospective Investigation into Cancer and Nutrition. Eur J Epidemiol 17: 1047-1053.

28. Mellstrom D, Johnell O, Ljunggren O, Eriksson AL, Lorentzon M, et al. (2006) Free testosterone is an independent predictor of BMD and prevalent fractures in elderly men: MrOS Sweden. J Bone Miner Res 21: 529-535.

29. Colditz GA, Hankinson SE (2005) The Nurses' Health Study: lifestyle and health among women. Nat Rev Cancer 5: 388-396.

30. Hunter DJ, Kraft P, Jacobs KB, Cox DG, Yeager M, et al. (2007) A genome-wide association study identifies alleles in FGFR2 associated with risk of sporadic postmenopausal breast cancer. Nat Genet 39: 870-874.

31. Hankinson SE, Manson JE, Spiegelman D, Willett WC, Longcope C, et al. (1995) Reproducibility of plasma hormone levels in postmenopausal women over a 2-3-year period. Cancer Epidemiol Biomarkers Prev 4: 649-654.

32. Tworoger SS, Missmer SA, Barbieri RL, Willett WC, Colditz GA, et al. (2005) Plasma sex hormone concentrations and subsequent risk of breast cancer among women using postmenopausal hormones. J Natl Cancer Inst 97: 595-602.
